# Contaminant Discharge From Outfalls and Subsequent Aquatic Ecological Risks in the River Systems in Dhaka City: Extent of Waste Load Contribution in Pollution

**DOI:** 10.3389/fpubh.2022.880399

**Published:** 2022-05-26

**Authors:** Nehreen Majed, Md. Al Sadikul Islam

**Affiliations:** Department of Civil Engineering, University of Asia Pacific, Dhaka, Bangladesh

**Keywords:** environmental and human health, aquatic system pollution, ecological risk, environmental contaminants, waste load, outfalls

## Abstract

Dhaka, the capital city, which is the nerve center of Bangladesh, is crisscrossed by six different rivers. A network of peripheral rivers connects the city and functions as a natural drainage system for a massive amount of wastewater and sewage by the increased number of inhabitants impacting the overall environmental soundness and human health. This study intended to identify and characterize the outfalls along the peripheral rivers of Dhaka city with the assessment of different pollution indices such as comprehensive pollution index (CPI), organic pollution index (OPI), and ecological risk indices (E_RI_). The study evaluated the status of the pollution in the aquatic system in terms of ambient water quality parameters along the peripheral rivers due to discharge from outfalls with a particular focus on waste load contribution. Among the identified outfalls, the majority are industrial discharge (60%), and some are originated from municipal (30%), or domestic sewers (10%). Water quality parameters such as suspended solids (SS), 5-day biochemical oxygen demand (BOD_5_), and Ammoniacal Nitrogen (NH_3_-N) for most of the peripheral rivers deviated by as much as 40–50% from industrial discharge standards by the environment conservation rules, Bangladesh, 1997. Based on the CPI, the rivers Buriganga, Dhaleshwari, and Turag could be termed as severely polluted (CPI > 2.0), while the OPI indicated heavy organic pollutant (OPI > 4) contamination in the Dhaleshwari and Buriganga rivers. The associated pollution indices demonstrate a trend for each subsequent peripheral river with significant pollution toward the downstream areas. The demonstrated waste loading map from the outfalls identified sources of significant environmental contaminants in different rivers leading to subsequent ecological risks. The study outcomes emphasize the necessity of systematic investigation and monitoring while controlling the point and non-point urban pollution sources discharging into the peripheral rivers of Dhaka city.

## Introduction

Water has been established as a significant source of myriads of services since it is required for the survival of all living species (1). The majority of the world's civilizations are inextricably linked to river water, where all civilizations began and flourished. The rivers and tributaries typically support a diverse range of biodiversity and create a diverse ecosystem comprised of ecologically sensitive and interconnected chemical, physical, and biological elements (2). For the manufacturing industries (like Dying, Garments, etc.), agricultural sectors, households, transport and communication, moreover for many living species, the river is a vital resource of water. On the other hand, Humans and other living creatures abound along the river's course. However, anthropogenic activities have been deteriorating river water in Bangladesh, making it unfit for human consumption or other uses (3). Some other causes of concern are water quality, particularly surface water, which is essential for drinking, fishing, agricultural, and industrial uses (4). Anthropogenic activities such as excessive urban development, uncontrolled industrialization, inadequate effluent treatment, and population growth have all caused significant concerns to the aquatic environment. As a consequence of the degradation of water quality, the aquatic environment is harmed, and the water becomes unsuitable for human consumption (5, 6).

With more than 230 primary. and minor rivers running throughout the country, Bangladesh is a low-lying riverine nation (3, 7), and Dhaka, which is the capital of Bangladesh, is shaded and connected through six different rivers. Being one of the fastest-growing capital cities, Dhaka is experiencing industrialization along the banks of the rivers. Because of the easy access to dumping facilities, and consequently, most water-contaminated regions are located in these industrialized districts (8). Because of the propensity for significant ecological and human health problems, such contaminated river water is unsuitable for human consumption, fishing, and agriculture (9). As per the findings, the physicochemical characteristics of water and relative environmental damage level in Dhaka's surrounding rivers have significantly deteriorated in terms of water quality indicators (10). Multiple industrial facilities, particularly Garment industries, have been developed in the current decades in Dhaka district's Savar Upazila, primarily along the Dhaleshwari river's bank. Garment industries are perhaps the most significant contributors among all of them together, accounting for 82% of total export income (28 billion USD/year) (11). Numerous industrial operations developed in the Hazaribagh region along the Buriganga river, including dyeing, textiles, batteries, and glass businesses. As a result of industrial activities, industrial pollution and effluents, including diverse environmental pollutants, significant waste loads are being contributed into the neighboring water bodies of Dhaka City. Furthermore, agricultural wash and urban municipal wastewater aggravate the potential risk associated with river water contamination. Heavy metals including Cadmium, Mercury, Lead, Copper, and Zinc are recognized important marine pollutants because of their toxicity, presence in food chains, and propensity to survive in the environment for an extended period of time (12, 13). Leather manufacturing involves many chemical products such as chromium sulfate, tannins, bactericides, and ammonia salt (14). The heavy metals may find their way into ecosystems and contribute contaminants of non-degradable nature. As a result, these heavy metals like Cadmium (Cd), Lead (Pd), and Zinc (Zn) continue to exist in the ecological system and pose a risk to humans and other animals (15). The heavy metals incorporate into the water body from anthropogenic sources though the protracted discharge of untreated or partially-treated waste, whereas metals are also introduced into agricultural land through the use of fertilizers and pesticides (16). Accumulation of metals in sediments and water at a significant quantity allows these metals to eventually enter the food chain *via* water and vegetation (17). In aquatic systems, heavy metals limit the generation of reactive oxygen species (ROS), affecting fish and the other aquatic creatures (18). These heavy metals are problematic because of their non-degradability; upon entering the ecosystem, they persist for a long time (19). Moreover, their distribution and accumulation in the aquatic ecosystem is a significant factor of concern due to the poisonous and pervasive nature of the metals. Which may create severe difficulties due to their ability to accumulate in live creatures and be bioaccumulated at relatively high trophic concentrations (20, 21). As a result, there is a high risk of river water contamination in Bangladesh's capital, which might have severe consequences for the riverine ecology and nearby residents by producing health problems from immediate consumption, ingestion and dermal exposure (22).

In response to such a demanding situation, supervision and assessment of surface water quality have become an international obligation (23). In emerging nations, maintaining sanitary systems is falling behind the speed of development and urbanization. Therefore, the current study aimed to analyze subsequent aquatic ecological threats in river systems of Dhaka City. This analysis also depicts the contribution of waste load to pollution in terms of discharge from outfalls. However, no comprehensive scientific investigation of waste load contribution toward pollution in the surface water of Dhaka's river systems as a whole has been published. A particular focus of this study consists of evaluating different pollution indices such as comprehensive pollution index (CPI), organic pollution index (OPI), and ecological risk index (E_RI_). To assess the state of contamination in the aquatic system in terms of ambient water quality indicators along peripheral rivers caused by outfall discharge. This would pave the way for and compel strategies to reduce the intensity and contribution of toxicity from outfalls into the rivers.

## Article Types

Original Research—Special Topic.

### Study Area

**Figure 1:**
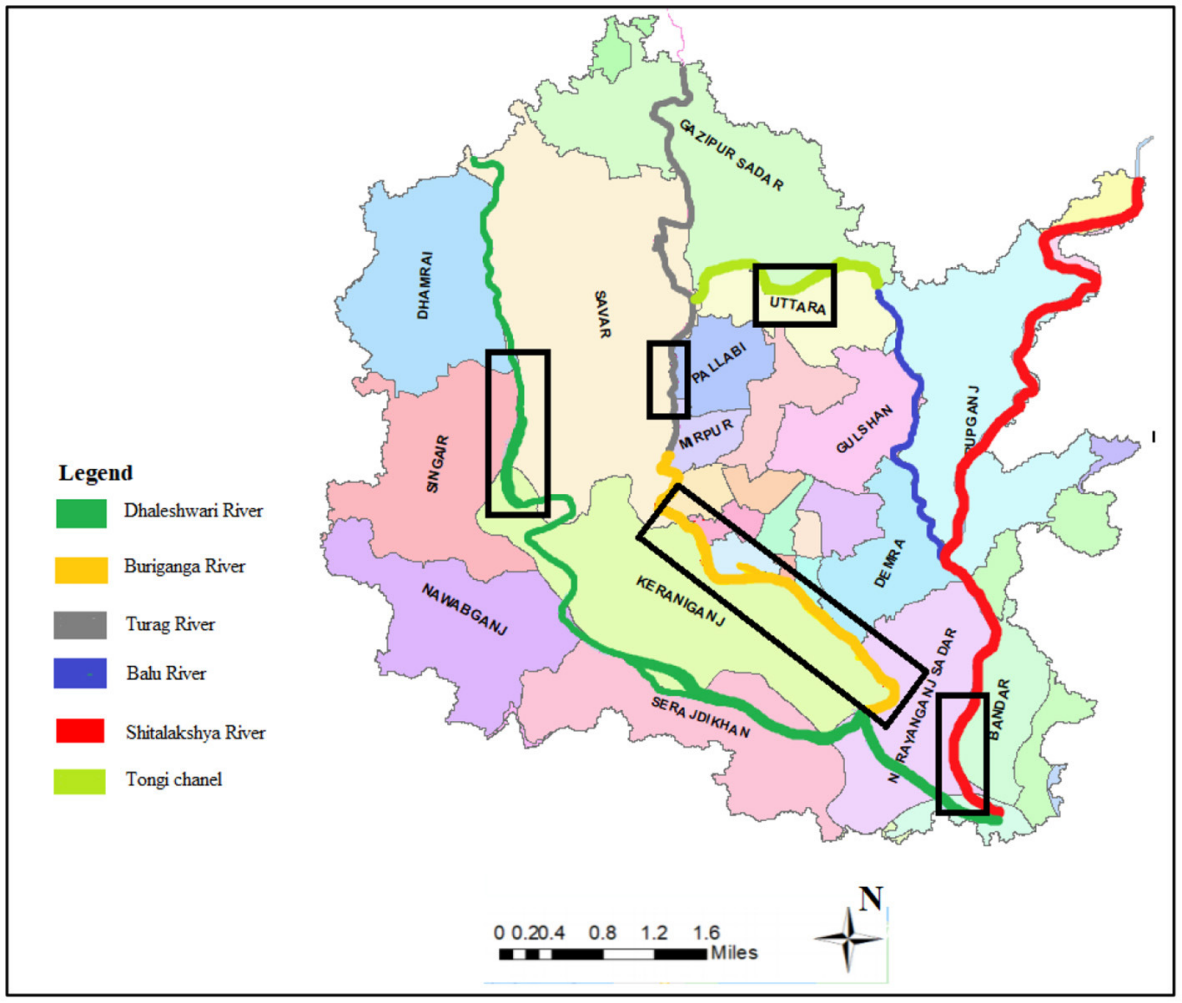
GIS map showing peripheral rivers around Dhaka watershed (boxes representing the sampling stretches along the rivers).

The river system of Dhaka city is primarily composed of three distinct systems: the Balu-Lakhya River System, the Bangsi-Turag-Buriganga-Dhaleshwari River System, and the Dhaleswari-Kaliganga River System (24) (Figure 1). To the west of Dhaka, the Dhaleswari-Kaliganga River and Bangsi- Shitalakshya-Turag-Buriganga River systems are located, while the Balu-Lakhya River system is located to the east. The Dhaleshwari River begins in the Jamuna River at the Tangail district's northwestern border and finally flows into the Shitalakshya River around the Narayanganj district (10). The Dhaleshwari River is one of the main tributaries of the Jamuna River in Dhaka, central Bangladesh, which is 160 km long and has an overall depth of 37 m (10). The Buriganga River system is situated in Bangladesh's North Central Zone's southeastern region, near the Padma (Ganges) and Upper Meghna rivers. The Buriganga River is a branch of the Dhaleswari River, the second largest river in the North Central Region, just after the Old Brahmaputra River. In fact, the Buriganga River is not inaccessible from a hydrological perspective, as previously stated. Around Dhaka, the Buriganga River has an average width of over 500 m and a length of almost 27 km (25). The majority of the Buriganga's water flow comes from the Turag River, which collects runoff from nearby rains and spillover from the Jamuna's left bank. The Turag River, which originates in the neighboring district of Gazipur, is about 63 km long (26). Between the middle forest and the Old Brahmaputra, the Lakhya River drains a substantial catchment area. A distributive tributary of the Brahmaputra, the Shitalakshya River travels in a northwesterly direction. Later, it makes a diversion to the east of Narayanganj and ends up at Kalagachhiya, where it joins the Dhaleshwari river. Near Narayanganj, this river has an average width of 300 m and a length of 110 km (27). A smaller catchment to the west of the Lakhya River feeds the Balu River; the Ichamati and Karnatali Rivers, which transport mostly overflows from the Padma and Jamuna Rivers, respectively, also contribute to the system's intakes (24). Tongi canal connects the Turag and Balu rivers on the western side, and the length of the canal is ~15 km (28). Buriganga, Shitalakshya, Turag, Tongi Canal, and Dhaleshwari rivers were chosen for the study to explore the aquatic ecological threats in Dhaka City's River systems. Figure 1 depicts the peripheral waterways system surrounding Dhaka Watershed, with the black boxes indicating the sampling sites from the rivers that are shown. Comprehensive identification of outfalls along each of the mentioned rivers was accomplished in terms of location and type which were then plotted on digitized map of the Dhaka Whatershed. For pollution study, outfalls on each of the rivers were selected according to the density of industrialized areas and the availability of garbage dumping stations along the banks of the rivers. Detailed figures for sampling locations and identified outfalls along each of the rivers are provided separately in the Supplementary Material.

### Identification of Outfalls and Collection of Sample

Outfalls from five distinct rivers were chosen for the current study, with 41 selected outfalls in total. The exact location of each sample site was determined using Global positioning system (GPS) data. The types of the outfalls and the locations are provided in Supplementary Material.

In total, 41 samples were collected from selected outfalls to analyse water quality parameters. From 27 km of primary tributaries of the Buriganga River, 24 outfalls were selected for present study from Aminbazar to Fatullah (Narayanganj), which covered 24 km of the river. Such comprehensive sampling was done for Buriganga river due to the densely located industries along the bank of the river. While six outfalls were assessed for Shitalakshya River from Kadamrasul to Mukterpur, the selected stretches encircled about 10 km of the river. For Turag River and Tongi Canal, three outfalls were chosen for each of the rivers, and each river covered 5 and 4 km, respectively. Furthermore, five outfalls were selected from about 8 km stretch for the Dhaleshwari River. Starting from the Savar Tannery area, the selected stretch of the Dhaleshwari River ended at Nama Bazar. Five additional samples were obtained from the Dhaleshwari River for heavy metals analysis. From the middle of the river's course, unfiltered samples of water were gathered. Following that, the samples were put in 100 mL polypropylene bottles and sealed. Before sending the samples to the University of Dhaka's Department of Soil, Water, and Environment Laboratory for heavy metals analysis, 1 mL of ultrapure nitric acid was added to each polypropylene bottle to produce a pH ~ 1 (29). The standard sampling protocol was performed for all the samples at each sampling site (30).

### Analysis of Water Quality Parameters

Water samples collected from all the rivers were analyzed in the Environmental Engineering Laboratory, Department of Civil Engineering, University of Asia Pacific for water quality characteristics. Total dissolved solids (TDS) concentrations were determined using DO700 EXTECH (Łódz, Poland) standard equipment (10). Electrical conductivity (EC), and total suspended solids (TSS) were measured with an EZDO (Taipei City, Taiwan) model “CTS-406” meter. A Twin (Santee, USA) model “B-221 pH” pH meter and a model “YK-22DO” dissolved oxygen meter were used to measure pH and dissolved oxygen (DO) (EZDO, Taipei City, Taiwan) (10) respectively. The 5-day biochemical oxygen demand (BOD_5_) was measured using the BODTRAK technique with a BOD Trak II (Model: Hach) and a BOD incubator (Model: Hach FOC120E) with potassium hydroxide and BOD nutrient buffer pillow reagents (31). The chemical oxygen demand (COD) was determined using a COD reactor (Model: Hach DRB200) and a spectrophotometer (Model: Hach DR 6000) through the reactor digestion technique (32). Colorimetric vanadomolybdophosphoric acid was used to detect phosphate (10). The colorimetric approach was used to assess nitrite by forming a reddish-purple azo dye at pH 2.0–2.5 by combining diazotized sulfanilamide with N-(1-naphthyl)-ethylenediamine dihydrochloride. A Shimadzu (Model: 1800 UV-Vis) spectrophotometer was used in colorimetric procedures (10).

### Analysis of Heavy Metals

Heavy metals, including cadmium (Cd), lead (Pb), and zinc (Zn) were analyzed in the Department of Soil, Water, and Environment, University of Dhaka. Shimadzu's (Model: AA-7000, South San Francisco, USA) atomic absorption spectrometer was used to determine the dangerous metal concentrations. All measurements were carried out using a precise (Model: ABS 220-4, Ziegelei, Balingen, Germany) precision electrical balance produced by KERN. A nylon membrane filter (47 mm diameter, Whatman, Washington, USA) was used (33). Each sample was obtained into a Pyrex volumetric flask containing 100 mL for heavy metal analysis. Following that, 9 mL of 1 M HCl and 3 mL of 1 M HNO_3_ were added. To lower the moisture content of the volumetric flask, it was gently heated in a sand bath under a fume hood. After the flask had been brought to room temperature, deionized water was poured. The filtrate was collected in a 250 mL HDPE screw-cap plastic container tube with a polypropylene/low-density polyethylene (LDPE) coated lid; Thermo Scientific, Washington, USA (10). Last but not least, a small number of samples were saved for use in calculating metal concentration. Different reference concentrations were used to calibrate the Atomic Absorption Spectrometer (AAS) for all metals. The average of three separate measurements was calculated for each data point. The detection limit was set at 0.001 mg/L in this study. In order to determine the level of metals in the sample, an oven was employed (Model: GAF-7000, ESCO, Changi South Street, Singapore).

### Comprehensive Pollution Index (CPI) and Organic Pollution Index (OPI)

Using monitoring data, the Comprehensive Pollution Index (CPI) determines the pollution level of a water body (34). Previously, Zaghden et al. (35) have also evaluated CPI to assess the ecological threats and status of discharge. The formula to calculate CPI is presented as follows:


$$\begin{array}{l} \text{CPI}=\frac{1}{n}\sum_{i=\;1}^{n}{\text{PL}}_{\text{i}} \end{array}$$

Where CPI is the Comprehensive Pollution Index; n is the number of variables under observation; PI_i_ is the pollution index number of ith observation. PI_i_ is calculated according to the following formula:


$$\begin{array}{l} {\text{PI}}_{\text{i}}=\frac{\text{Ci}}{\text{Si}} \end{array}$$

where C_i_ is the measurement of parameter's concentration in water and S_i_ is the allowable number of parameters in accordance with environmental standards. Mishra et al. (36) classified CPI into five categories (provided in the Supplementary Material) which was utilized to evaluate the pollution categories relevant for the rivers based on the estimated values of CPI for the outfalls discharging into the rivers.

OPI is a tool for assessing a watershed's pollution intensity depending on four distinct characteristics such as dissolved inorganic phosphate, COD, and dissolved inorganic nitrogen (2), as well as the concentration of dissolved organic carbon (DIP). Dou et al. (37) analyzed OPI to evaluate environmental risks from sewage discharge in urban area. The following equation represents the organic pollution index (OPI):


$$\begin{array}{l} \text{OPI}=\frac{\text{COD}}{\text{CODs}}+\frac{\text{DIN}}{\text{DINs}}+\frac{\text{DIP}}{\text{DIPs}}+\;\frac{\text{DO}}{\text{DOs}} \end{array}$$

According to the environmental standard, CODs, DOs, are the standard concentrations of COD, and DO; DINs are the total restricted concentration of Nitrate, Nitrite, and Ammoniacal Nitrogen; and DIPs are the limited concentration of Phosphate.

According to the OPI value, water quality could well be categorized into six different levels according to (38) which are provided in Supplementary Material. OPI values were evaluated for OPI based categorization of risk from all the outfalls discharging into the respective rivers.

### Assessment of Ecological Risk Index

A sedimentological technique proposed by Hakanson (39) might be used to first identify how heavy metal contaminants behave naturally and environmentally. Toxic response indicators, a precise pollution measurement, and a probable ecological risk index are all incorporated in the process of determining pollution coefficients. The following equations yielded the ecological risk index (E_RI_) for the study area (39):


$$\begin{array}{lll} E_{\text{r}}^{\text{i}} & = & T_{\text{r}}^{\text{i}}\;\left(C^{\text{i}}/C_{\text{o}}^{\text{i}}\right)\\ E_{\text{RI}} & = & \sum E_{\text{r}}^{\text{i}} \end{array}$$

Where C^i^ and ${\text{C}}_{\text{o}}^{\text{i}}$ denotes the amounts of specific heavy metals and their allowable reference value, respectively, and ${\text{E}}_{\text{r}}^{\text{i}}$ denotes an ecological risk factor. Each metal has a different toxicity factor (Cd = 30, Pb = 5, and Zn = 1) which is referred as ${\text{T}}_{\text{r}}^{\text{i}}$ (40). The ecological risk index (E_RI_) quantifies the sensitivity of biological populations to certain metals in the region under consideration. **Table 4** shows the ranges of the indices of ${\text{T}}_{\text{r}}^{\text{i}}$ and E_RI_ based on which the categorization of risk was evaluated for the outfalls discharging into the rivers. Li et al. (41) also analyzed Ecological Risk Index to evaluate subsequent ecological threats from industrial wastewater discharge. Accordingly, the present study accomplished the categorization of ecological danger associated with hazardous metals in the selected outfalls following the Classification of Ecological risk index. Furthermore, E_RI_ of heavy metal pollution (42) which is provided in the Supplementary Material.

### Waste Loading Estimation

The authors estimated the flow rates of the rivers Buriganga, Shitalakshya, Turag, Tongi Canal, and Dhaleshwari according to the procedure described in Alam et al. (43) which determined the waste loading rate using a distance technique rather than particular flow measurement equipment. The first step was to determine the cross-sectional area of the outfall. Then, using a specific distance, the velocity of outfalls was determined. The cross-sectional flowing area was multiplied by the measured discharge speed to get the flow rate. Direct measurements of the flow rates at all sample outfalls were made during the field visit. Following the flow measurement, the waste loading rate of the particular pollutant was determined by following equations which yielded the waste load for the study area (44):


$$\begin{array}{l} \text{Wasteload}=\text{Flowrate}{\;}^{*}\text{ Concentration} \end{array}$$

Where, Flow rate represents the flow rate of particular outfall and Concentration denotes level of concentration of a specific pollutant. Waste loads were estimated for the parameters including 5-day biochemical oxygen demand (BOD_5_), chemical oxygen demand (COD), ammoniacal nitrogen (NH_3_-N), total suspended solids (TSS), total dissolved solids (TDS), and electrical conductivity (EC). Digitized waste load maps were prepared to demonstrate the waste load contribution of the outfalls in the selected rivers.

## Results

### Identification of Outfalls Along Dhaka Watershed

Several industrial outfalls (tanneries, dyeing, textiles, power plants, etc.), storm sewer outfalls, and domestic outfalls have been identified in the Dhaka Watershed for the present study. Runoff from streets, wastewater from marketplaces, vehicle workshops, clinics, hospitals, and other outfalls bring in contaminants from several sources. Aside from that, the Buriganga River has four known illegal storm sewage outfalls (box culverts). Field surveys, analysis of available maps (on the drainage of Dhaka City and storm sewer network), and discussions with officials of the Dhaka Water Supply and Sewerage Authority (DWASA), which is responsible for managing both domestic sewage and stormwater drainage, were used to identify outfalls along the Dhaka Watershed. Table 1 depicts the identified outfalls along with significant parts of the river stretches of Dhaka Watershed. A thorough inventory of outfalls was compiled (including information on outfall location, type of discharge, and the number of outfalls) and is reported in the current study. This observation of outfalls showed that nearby industrial sources heavily influenced surface water quality indicators. Comprehensive identification of outfalls revealed that the majority of the outfalls (around 60%) are industrial discharge, and some are originated from municipal (just below 30%) or municipal sewers (near about 10%).

**Table 1:** Identified outfalls along the river stretches of Dhaka Watershed.

**Rivers**	**Type of discharge**	**Number**	**Outfall location**
Dhaleshwari River	Tannery	8	Kolatoli and Hemayetppur
	Dyeing	6	Savar
	Garments	15	Savar
	Power plant	3	Narayanganj
	Jute mill	4	Narayanganj
	Cement factories	2	Narayanganj
	Municipal sewer	8	Savar and Sudkhira
Buriganga River	Power plants	2	Fatullah
	Garments	5	Fatullah
	Tannery	10	Hazaribagh Area
	Dyeing	3	Shyampur and Postogola
	Storm sewage	4	Hazaribagh Area and Shyampur
	Municipal sewer	10	Shyampur and Postogola
Shitalakshya River	Cement	8	Narayanganj and Fatullah
	Garment	14	Narayanganj and Fatullah
	Jute mills	7	Narayanganj and Fatullah
	Power plants	5	Narayanganj and Fatullah
	Dyeing	2	Narayanganj and Fatullah
	Municipal sewer	10	Narayanganj and Fatullah
Turag River	Garments	5	Kaundiya
	Dyeing	2	Diabari Ghat
	Municipal sewer	4	Miepur Bridge Road
Tongi Canal	Garments	15	Bismillah Market
	Dyeing	3	West Abdullapur
	Municipal sewer	3	Tongi Bridge

The majority of the outfalls were located in areas with high industrial disposals, agricultural activity intensities and numerous sources of pollution, both point and non-point. Additionally, several of the discharge points serve as municipal supplies of water. Point sources include a variety of Industrial fields, including leather, Dying, Textiles, and Metals manufacturing. Industrial processes such as the production of textiles, inks, batteries, and metal melting furnaces are also considered point sources of pollution. Point sources such as garbage disposal sites, toxic sewage, ports, and landing stations are all contributing factors to pollution. The figure demonstrating the identified outfalls in Dhaka Watershed as digitized have been provided in the Supplementary Material.

### Assessment of Water Quality Parameters of Outfalls

Water samples from the outfalls of the rivers of Dhaka city were examined for several water quality parameters. Using the Environmental Conservation Rule, Bangladesh (ECR'97), assessment of outfall discharge quality was made and averaged for the respective rivers which are summarized in Table 2.

**Table 2:** Results of water quality parameters of the selected outfalls from peripheral rivers of Dhaka City.

**Parameter**	**Unit**	Standard^*^	**Average** **±standard error of mean**				
			**Dhaleshwari River (*n* = 5)**	**Turag River (*n* = 3)**	**Tongi Canal (*n* = 3)**	**Buriganga River (*n* = 24)**	**Shitalakshya River (*n* = 6)**
pH		6–9	9.1± 1.8	8.28 ± 0.48	7.28 ± 0.93	7.12 ± 0.35	6.52 ± 0.71
TSS		150	424.8 ± 165.8	131 ± 1.41	131 ± 1.41	242 ± 93.11	298.33 ± 153.88
TDS	mg/L	2,100	2,325.8 ± 368.2	606 ± 72.12	606 ± 69.29	561.08 ± 94.57	661.17 ± 157.7
BOD_5_	mg/L	50	86.72 ± 30.8	128.4 ± 7.07	88.4 ± 7.07	181.9 ± 31.86	126.2 ± 32.63
COD	mg/L	200	486.6 ± 114.5	1,222.8 ± 184.9	722.8 ± 43.55	549.41 ± 157.32	306.17 ± 142.28
DO	mg/L	4.5–8	0.52 ± 0.6	1.14 ± 0.83	1.49 ± 0.08	1.43 ± 1.51	0.85 ± 0.52
NH_3_-N	mg/L	50	71.96 ± 19.8	50.3 ± 4.52	35.3 ± 2.54	44.88 ± 33.53	25.15 ± 17.9
EC	μS/cm	1,200	2,277.8 ± 191.3	1,512 ± 438.40	1,412 ± 155.56	1,271.17 ± 176.48	1,386.5 ± 262.86

* *Standards for waste from industrial units or projects waste: the environment conservation rules, Bangladesh, 1997 (ECR'97), n = number of outfalls*.

**Figure 2:**
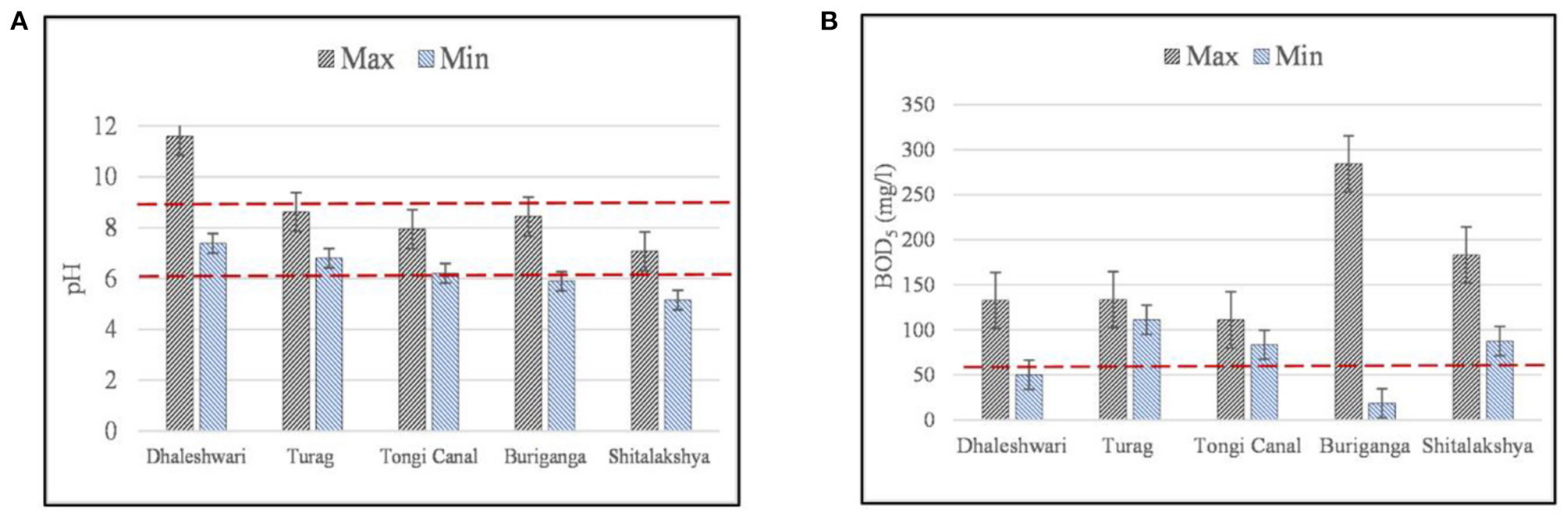
**(A,B)** Maximum and minimum concentrations of pH and BOD_5_ for the selected outfalls along the peripheral rivers of Dhaka (error bars indicate standard error of mean and red dotted lines indicate the Bangladesh standards of water quality according to ECR'97).

It is shown in Tables 1, 2 that how the combined discharge from several point sources are contributing together from outfalls at many locations. With ECR'97 standards in red dotted lines, Figures 2A,B show the maximum and minimum levels of pH and BOD_5_ respectively for the selected outfalls along each of the rivers in Dhaka City.

Dhaleshwari, Buriganga, Shitalakshya, and Balu rivers, in particular, were discovered to be black in color visually and were experienced with unpleasant smells during the visual investigation. With pH levels ranging from 7.38 to 11.6 for the Dhaleshwari River outfalls. The maximum pH value was observed at outfall D-1 (Savar Tannery), and the minimum pH value was recorded at outfall D-4 (AKS dying). Except for Dhaleshwari for maximum level (pH = 11.6) and Shitalakshya for minimum level (pH = 5), all of the other outfalls on the rivers in Dhaka Watershed were found to have pH values within the acceptable range for this study (Figure 2A). The most acidic outfall of all the Peripheral Rivers in the Dhaka region was observed in the Shitalakshya River.

Organic contamination may best be assessed using BOD_5_ analysis, the standard for this kind of analysis (45). The BOD_5_ concentration of the outfalls of Buriganga River varied between 18.4 and 284.2 mg/L from 24 outfalls. Indicating that there is significant variation of the organic content among the outfalls while the maximum value indicates the highest discharge level among all the rivers making it the most polluted of all the rivers under study (Figure 2B). Similarly, the observed average BOD_5_ values of the outfalls in Dhaleshwari river (86.72 mg/L), Turag River (128.4 mg/L), Tongi Canal (88.4 mg/L), and Shitalakshya River (126.2 mg/L), respectively, exceeded the BECR guidelines (50 mg/L) for the permissible limit of BOD_5_ (Figure 2B). The BOD_5_ standard for discharge from public sewerage system connected to treatment at the second stage is 250 mg/L, and that for irrigated land is 100 mg/L (46). Since the outfalls pass through a densely inhabited and industrialized sector along the riverbanks, the BOD_5_ concentration was more significant around the particular periphery of the waterways segment of Dhaka city. A variety of organic and chemical pollutants can build up in the waterways because of the discharge of organic materials due to the inefficiency of sewage treatment plants, stormwater runoff, agricultural slurries, domestic waste (food and human waste), industrial waste (waste from food processing, tanning, and dying), and silage liquor. There is a consistent, similar rate of discharge of organic compounds and resulting contamination in all of the surrounding rivers, as shown by this observation.

**Figure 3:**
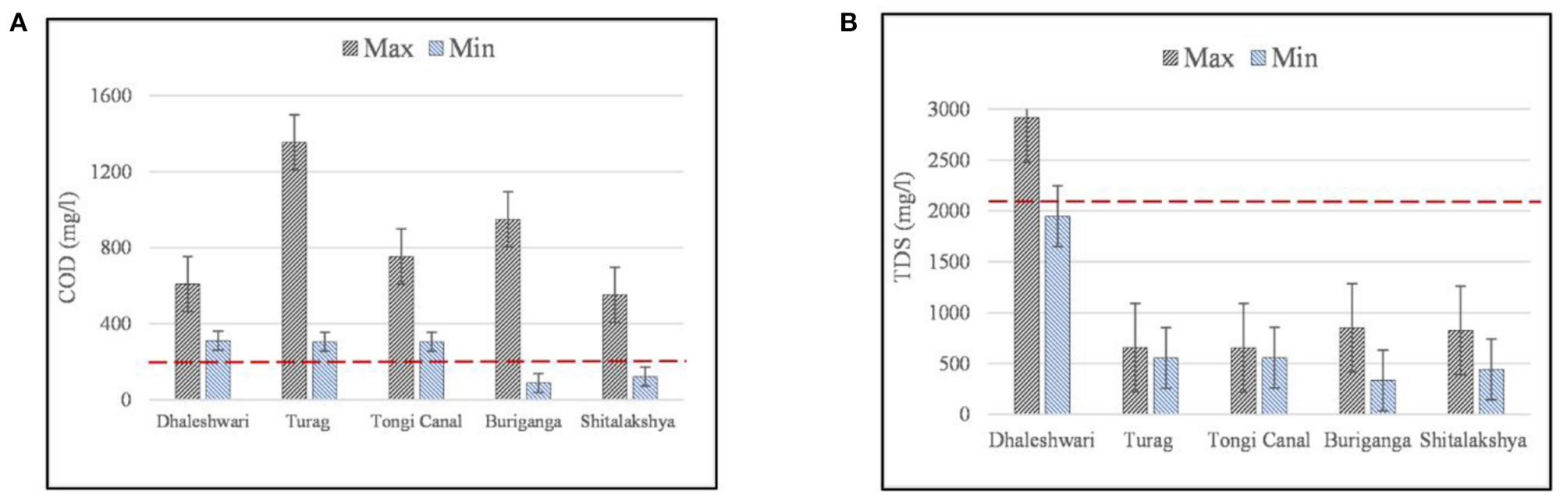
**(A,B)** Maximum and minimum concentrations of COD and TDS for the selected outfalls along the peripheral rivers of Dhaka (error bars indicate standard error of mean and red dotted lines indicate the Bangladesh standards of water quality according to ECR'97).

With ECR'97 standards in red dotted lines, Figures 3A,B shows the maximum and minimum levels of COD and TDS, respectively, for the outfalls along the rivers in Dhaka City. The capability of industrial waste and sewage to resist pollutants and the amount of oxygen needed to oxidize organic and inorganic components in a sample may both be determined using the COD method (chemical oxygen demand) (47). COD values ranged from 305–1,353.6 mg/L for the outfalls in Turag River, 305–753.6 mg/L for those in Tongi Canal, 89–949 mg/L for those in Buriganga River, 311–609 mg/L for the ones in Dhaleshwari River, and 122–552 mg/L for the ones in Shitalakshya River. The results are suggesting a high level of contamination in these rivers based on the ECR'97 guideline (200 mg/L) (Figure 3A). Turag River has the highest level of organic forms of discharge, as observed among all the rivers in Dhaka City. Outfalls with greater COD levels are more likely to include industrial pollutants comprising inorganic and organic chemicals, which indicates a higher toxicity level than samples with lower COD levels (48).

Minerals, alkalis, certain colloidal and dissolved solids in water, some acids, sulfates, metallic ions, etc., are all included in the total dissolved solids (TDS) category (49). TDS levels in the Dhaleshwari River water varied from 1,948 to 2,914 mg/L, with the highest level found at outfall D-1 (Savar Tannery) and the lowest level recorded at outfall D-2 (Sudkhira) (Figure 3B). Savar Tannery (D-1) is the only designated outfall in Dhaka City that exceeds the allowable level of ECR'97 standards for the discharge standard (2,100 mg/L), which discharges into the Dhaleshwari River system. When the TDS level reaches 1,000 mg/L, the water becomes murkier and saltier, which severely influences aquatic life (50, 51). As a result, humans, agriculture, and animals all are affected. However, during the monsoon season, runoff water flow may fluctuate while influencing the irrigation system. An increase in TDS levels has been linked to dyeing unit discharge in other dyeing-heavy locations (52).

**Figure 4:**
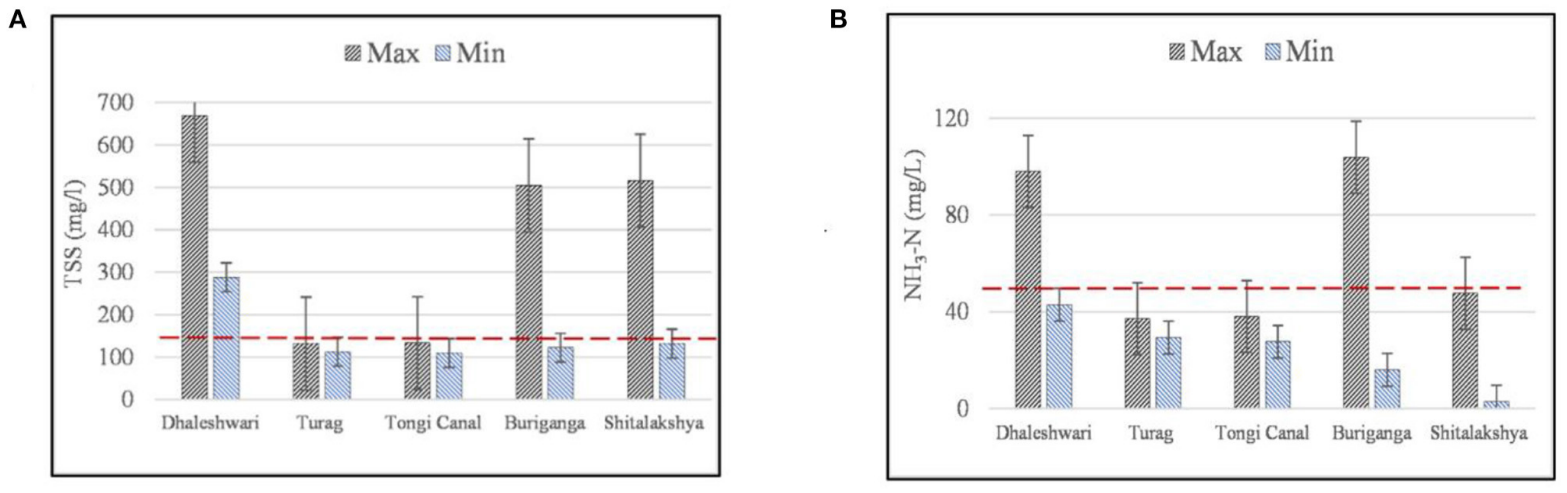
**(A,B)** Maximum and minimum concentrations of TSS and NH_3_ for the selected outfalls along the peripheral rivers of Dhaka (error bars indicate standard error of mean and red dotted lines indicate the Bangladesh standards of water quality according to ECR'97).

With ECR'97 standards in red dotted lines, Figures 4A,B shows the maximum and minimum levels of TSS and NH_3_-N, respectively, for the outfalls along the rivers in Dhaka City. Total suspended solids (TSS) levels in the outfall discharge along Dhaleshwari River varied from 288 to 669 mg/L, varied from 122 to 505 mg/L for Buriganga River and varied from 132 to 516 mg/L for Shitalakshya River (Figure 4A). The results descrived above are exceeding ECR'97 guidelines (150 mg/L) for the permissible limit of TSS discharge standard. As important as the analysis of BOD_5_ is the assessment of suspended particles in sewage and other wastewater investigations (53). To avoid putrefaction, it is best if there are no suspended solids in the canal. However, various organic compounds may also be present in the suspended particles.

The Ammoniacal Nitrogen (NH_3_-N) values in the outfalls along the Dhaleshwari River varied from 42.9 to 98 mg/L and varied from 16 to 103.8 mg/L for Buriganga River, exceeding ECR'97 guidelines (50 mg/L) for the permissible limit of NH_3_-N as shown in Figure 4B. Both maximum and minimum levels of NH_3_-N discharge levels were below the standard limits along Turag, Tongi Canal and Shitalakshya rivers. Industries along Buriganga and Dhaleshwari seem to be contributing toward elevated ammonia levels along the rivers in relevance to the inadequacy in their treatment of effluents.

**Figure 5:**
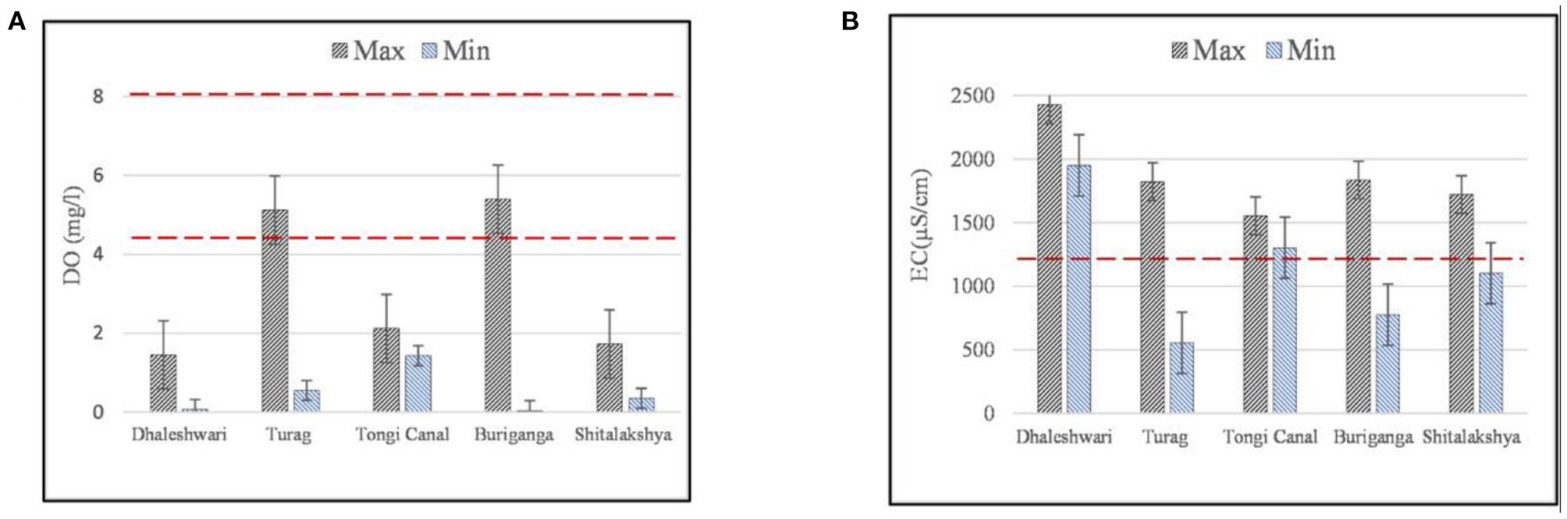
**(A,B)** Maximum and minimum concentrations of DO and EC for the selected outfalls along the peripheral rivers of Dhaka (error bars indicate standard error of mean and red dotted lines indicate the Bangladesh standards of water quality according to ECR'97).

With ECR'97 standards in red dotted lines, Figures 5A,B show the maximum and minimum levels of DO and EC for the outfalls along the rivers in Dhaka City. It is essential for aquatic species in surface waters to have a high quantity of dissolved oxygen (DO) (54, 55). Oxygen-depleting pollutants may be detected by decreasing dissolved oxygen (DO) concentration in the water body. Many water quality elements and processes, such as bacterial metabolism, algal photosynthesis etc., are influenced by the amount of dissolved oxygen available in the medium (56). DO levels varied within 0.07–1.45 mg/L for the outfalls in Dhaleshwari River, 0.04–5.4 mg/L for those in Buriganga River, 0.35–1.73 mg/L for those in Shitalakshya River, 0.55–5.12 mg/L for the ones in Turag River, and 1.43–2.12 mg/L for the ones in Tongi Canal (Figure 5A). For the waste discharged from industrial units into inland surface water, these levels should be within 4.5–8 mg/L, according to the ECR'97 recommendations adding enough DO in the river water. The lowest level of DO was obtained next to the Savar tannery area. The following DO criteria are permissible following the Environmental Quality Standard (EQS): Fish and domesticated animals need 4–6 mg/L; 6 mg/L for drinking, 4–5 mg/L for industrial purposes, and up to 5 mg/L for industrial applications (57). Organic chemicals released from sources such as wastewater treatment facilities, storm floods, slurry agriculture, and alcohol silage are possible explanations for the depleted dissolved oxygen levels in the water. Biodegradable waste from industrial and household sources has a rapid decrease in DO level by supporting microbes in the water body. Oxygen is essential for all aquatic organisms with aerobic respiration biochemistry to operate appropriately (58). The quantity of dissolved oxygen (DO) decreases when BOD_5_ levels are high since microorganisms consume the oxygen they acquire from the water (59). As a result, fish and other aquatic animals cannot thrive in oxygen-depleted environments.

EC varied from 554 to 1,822 μS/cm for the outfalls along the Turag River, 1,302 to 1,554 μS/cm for those in the Tongi Canal, 775 to 1,835 μS/cm for those in the Buriganga River, 1,102 to 1,722 μS/cm for the ones in the Shitalakshya River, and 1,950 to 2,428 μS/cm for those in the Dhaleshwari River (Figure 5B). According to WHO standards, rivers in these areas contain high amounts of ionic pollution. According to WHO guidelines, a body of water with an EC of more than 1,200 μS/cm is not acceptable for agriculture, home use, swimming, industrial use, or drinking. Electrical conductivity may have increased due to tannery and metal plating industry emissions. Heavy metals are also produced in the textile and dyeing industries. Plants and other organisms in the environment may be affected by high levels of EC, which may have a physiological impact (10). Water from industrial and municipal sources and effluent from sewage treatment plants have been shown to contain significant quantities of ionic pollutants, which may harm aquatic species.

### Comparative Assessment of Heavy Metal Contamination

The heavy metals concentrations in the outfalls along the Dhaleshwari River investigated are shown in Table 3. Additionally, this table includes the concentrations of heavy metals in theother outlying rivers of Dhaka, as reported in prior research. Furthermore, Table 3 also comprises ECR'97 discharge standard guidelines. However, when it came to heavy metal concentrations, Zn was at the highest level, followed by Pb and Cd.

**Table 3:** Heavy metals concentration (mg/L) levels in the outfalls of the Dhaleshwari River and the other selected peripheral rivers in Dhaka city.

	**Cd**	**Pb**	**Zn**	**References**
D-1	0.42	3.9	5.49	Present Study
D-2	0.25	0.63	1.6	Present Study
D-3	0.13	1.53	2.21	Present Study
D-4	0.21	0.49	2.33	Present Study
D-5	0.38	1.97	4.29	Present Study
Buriganga River	0.34	3.42	3.15	(60)
Shitalakshya River	0.025	1.112	3.12	(61)
Turag River	0.005	0.084	3.1	(62)
Tongi Canal	0.08	1.34	4.65	(63)
ECR'97^a^	0.05	1	5	(53)
^a^ECR'97 = The Environment Conservation Rules, Bangladesh				

a *Standards for waste from industrial units or projects waste: the environment conservation rules, Bangladesh, 1997 (ECR'97)*.

Savar tannery industrial zone had the most significant concentrations of Cd pollution at outfall D-1 (0.42 mg/L), whereas outfall D-3 (Sudkhira) (0.015 mg/L) in the Dhaleshwari River (0.015 mg/L) had the lowest concentration (Table 3). However, permissible levels of Environmental Conservation Rules (0.005 mg/L), World Health Organization (0.003 mg/L), and Food and Agriculture Organization (0.01 mg/L) were all surpassed in these experiments (64, 65). In the high Cd-containing region of the Savar District, one of the oldest and most popular wholesale fish markets is located. Higher levels of Cd might fluctuate with the capacity of river water, with the decreased flow of water promoting metals to precipitate in sediment, raising Cd concentrations (66). Tongi Canal and Shitalakshya river both demonstrated Cd concentrations of 0.02 and 0.025 mg/L, according to Sunjida et al. (63) and Haque (61). Cd levels in the Buriganga River were found to be as high as 1.34 mg/L in a previous study (60). Chromium-based chemicals might have contaminated the Buriganga River from cooling towers. Industrial activities, leachates from defused batteries, and Cd-plated materials might contribute to the high amount of Cd in the Buriganga River and Dhaleshwari River (67, 68). Although near the Savar tannery effluent zone along the selected stretches of the Dhaleswari River, the concentration of Cd seems higher than that in Buriganga, which is also above the acceptable limit. Because water cotyledons (*E. crassipes*) grew around the sample location during the investigation, these water cotyledons accumulated Cd and were dubbed as chrome-sorbent plants (69, 70).

Lead (Pb) is a significant contributor of pollution from sources connected to battery recycling plants, and it is also thought to be a good indicator of contamination from urban runoff water (71). Outfalls along the Dhaleshwari River showed varying levels of Pb ranging from 0.49 to 3.9 mg/L. According to the Environmental Conservation Rules, Bangladesh, the permissible level of Pb in drinking water is 1 milligram per liter [Table 3; (53)]. The primary sources of Pb in the urban area include municipal runoffs, untreated or poorly treated industrial effluents, atmospheric deposition (72) and similar activities observed along the Buriganga and Dhaleshwari river bank. Batteries, pigments, and plating enterprises are among the possible sources of Lead in the outfalls discharging into the Dhaleshwari River (73). The highest level of Pb was obtained at outfall D-1 (3.9 mg/L), which is greater than the ECR'97 permitted limit (1 mg/L), and quite possibly could be attributed to Savar tannery effluents. Except for D-2 and D-4, every outfall in the vicinity of Savar City's industrial district has a Pb concentration that exceeds the allowable limit. A long-term lead consumption exceeding the permissible level might induce allergic skin reactions (74). In Alexandria, Egypt, El-Ebiary et al. (69) observed that red tilapia mortality was induced by exposure to high levels of cadmium and Lead. Zn concentration levels in the selected rivers of Dhaka City are shown in Table 3. Zinc (Zn) concentrations in the outfalls along the Dhaleshwari River ranged from 1.6 to 5.49 mg/L (Table 3). Except for D-1, all of the outfalls in the Dhaleshwari River contained the maximum Zn concentration below the levels permitted by the ECR'97 (5 mg/L). Despite this, the present investigation found that the content of Zn in selected peripheral rivers in Dhaka city exceeded the water quality standard limit for Zn (5 mg/L) (23). In the Shitalakshya River, Haque (61) had reported an average (the arithmetic mean) concentration of Zn of 3.12 mg/L, while Jahan (60) found it to be 3.15 mg/L for the Buriganga River. The levels of discharge provide an indication of the heavy metal concentrations that can be potentially contributed from industries. Chronic exposure to zinc, a carcinogenic metal that damages the liver and heart and lowers metabolism and skin sensitivity, may even cause cancer (75).

### Characterization Based on Pollution Indices (CPI)

**Figure 6:**
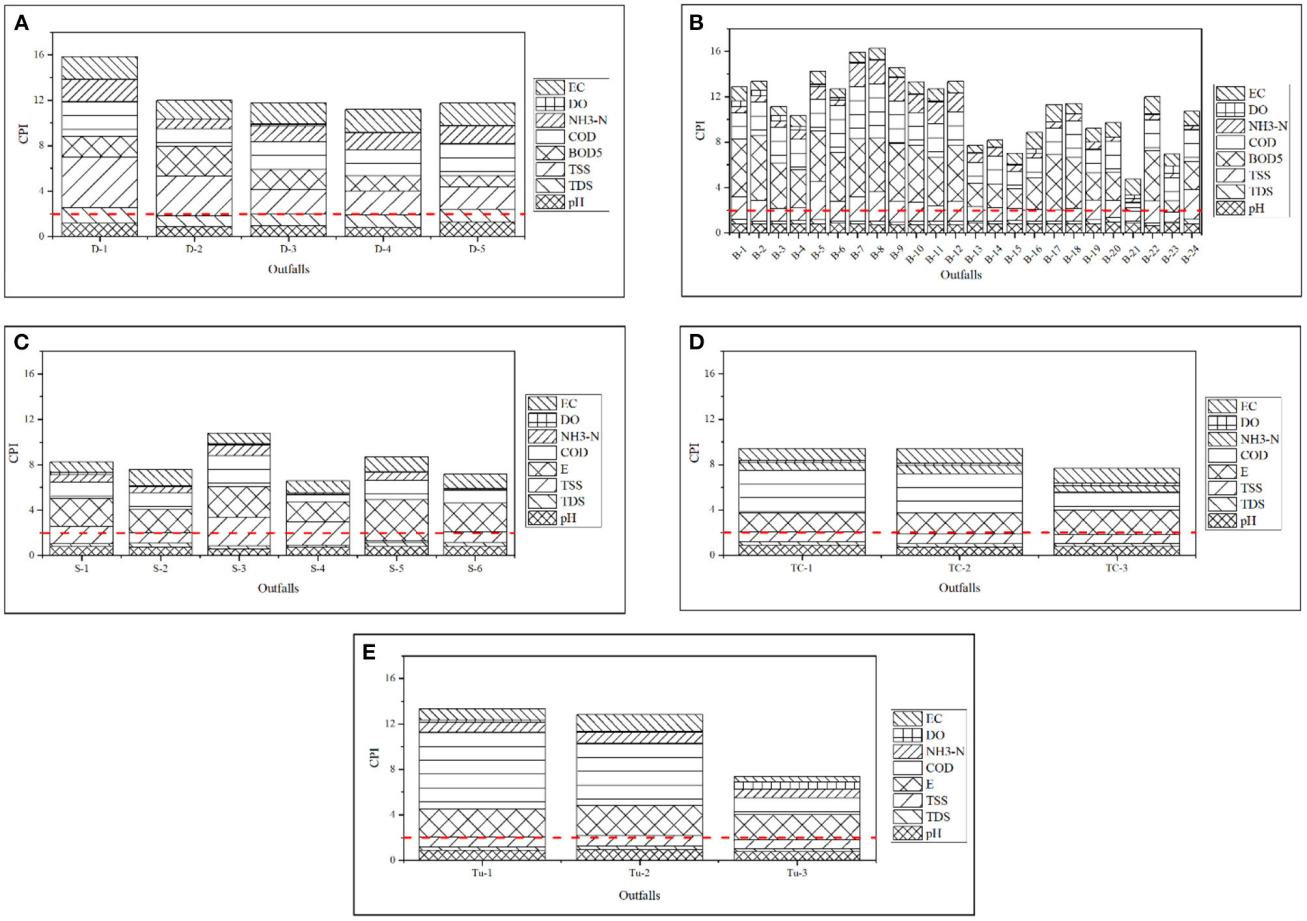
**(A–E)** Assessment of CPI for the outfalls in **(A)** Dhaleshwari River, **(B)** Buriganga River, **(C)** Shitalakshya River, **(D)** Tongi Canal, and **(E)** Turag River (dotted lines indicate a severely polluted category of CPI).

Using a simple numerical measurement, the comprehensive pollution index (CPI) can express the overall quality of the discharge and categorize it into numerous subcategories (76). This study uses the holistic and detailed pollution analysis technique to characterize the discharge quality of outfalls along the selected rivers of Dhaka Watershed to depict the river's quality based on single-factor analysis in a comprehensive manner. Using the ECR'97 standard limit as a guide, this research performed the Characterization based on Pollution Indices (CPI). Figures 6A–E illustrates the CPI ranges for water quality of identified outfalls in the respective rivers. According to CPI, scores vary from 4.73 to 16.29 for selected outfalls from the Dhaka Watershed, indicating that the Dhaleshwari, Buriganga, Shitalakshya, Turag, and Tongi Canal rivers are seriously contaminated (CPI ≥ 2) (Figure 6) and should not be used for irrigation. Due to substantial and direct inputs of industrial wastes from a Box culvert in the Hazaribagh region in the Buriganga River, the outfalls B-8 to B-10 (more than 2) have earned the highest score.

The present study discovered that the water of the selected river is inappropriate owing to its high COD and deficient DO levels as contributed by the outfalls. Consequently, it might show that the river's assimilation or resistance ability has been aggravated. Due to pollution caused by frequent human interventions, including cremation, sewage discharge, agricultural runoff, as well as detergents from textile washing and bathing (77). According to CPI: 11.2–15.84, the Dhaleshwari River has been highly contaminated. The CPI calculated for each sampling outfalls of Shitalakshya, Turag and Tongi Canal are also observed to be severely polluted (CPI ≥ 2.0). Compared to the other rivers under evaluation, the Buriganga River's water quality is shown to be typically poorer, whereas Tongi Canal was found to be less contaminated in terms of CPI. In general, the water quality of each river in the Dhaka Watershed has deteriorated over time due to increased anthropogenic pressure that exceeds the river's capacity for assimilation or tolerance (36).

### Organic Pollution Index (OPI)

**Figure 7:**
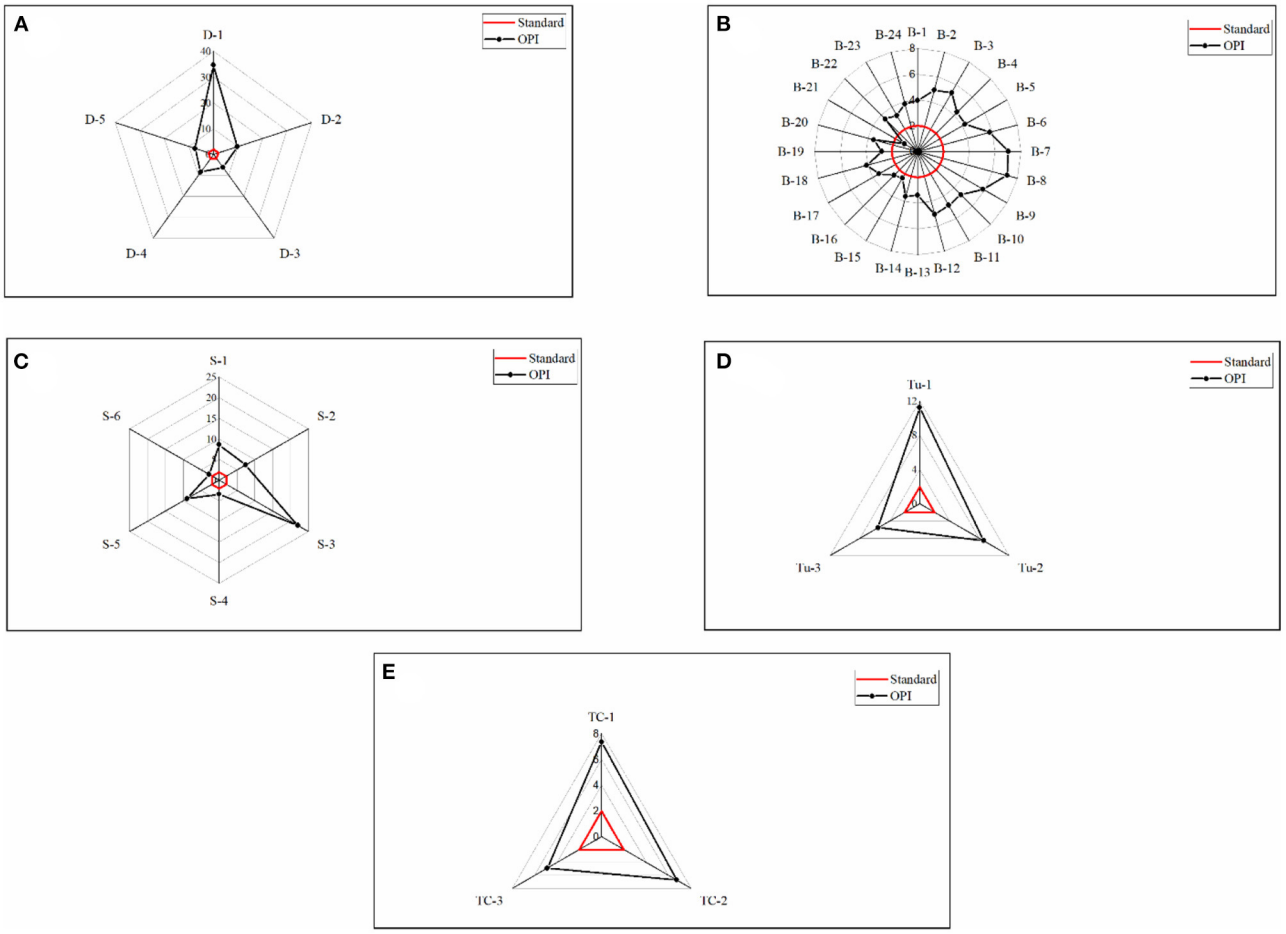
**(A–E)** Assessment of OPI in the outfalls of **(A)** Dhaleshwari River, **(B)** Buriganga River, **(C)** Shitalakshya River, **(D)** Tongi Canal, and **(E)** Turag River (red lines indicate a heavily polluted level of OPI).

The Organic Pollution Index (OPI) is a commonly used metric for determining the degree of organic pollution (78). Figures 7A–E illustrate the assessment of OPI from different identified outfalls of selected rivers through polar charts in Dhaka Watershed. The OPI of outfalls from the Dhaleshwari River water as obtained in this research varied between 6.18 and 34.69, which corresponds to the heavily polluted category (OPI ≥ 4) among all other rivers evaluated in this study (Figure 7A). This indicates that all sampling sites along the selected stretches of the Dhaleshwari River have a significant degree of eutrophication (79). Outfall D-1 (Savar Tannery) had the highest OPI (34.69), and outfall D-3 (Sudkhira) had the lowest OPI (6.18) values throughout the sampling timeframe. All outfalls have been thus classified as heavily polluted (OPI ≥ 4). It is possible that the increased absorption of nutrients by phytoplankton and aquatic plants is to blame for the higher OPI values (80). Such facilities that release organic pollutants straight into marshes with no prior treatment may be held responsible for the devastating effects they have, as this information shows. In addition to the influence of Industries and Dying plants that immediately drain wastewater to the rivers without treatment, there is also a low flow of water coming from the Dhaleshwari River into the marshes. As a result of this action to dilute wastewater, the rivers have been labeled as organically contaminated wetlands, which is thought to be the reason for their poor quality (81).

Among the outfalls along Shitalakshya River, outfall S-6 was found to be slightly polluted (2 ≤ OPI < 3), and outfall S-4 was observed as moderately polluted (3 ≤ OPI < 4). Figure 7 (polar charts) depicts that every selected outfall from Tongi Canal, Turag River and outfalls S-1, S-2, S-3, and S-5 of Shitalakshya could be classified as heavily polluted (OPI ≥ 4). Out of 24 outfalls in the Buriganga river, most of the outfalls are characterized by strong organic pollution (OPI ≤ 4) throughout the study period (Figure 7B). Outfall B-21 could be classified to be at the beginning of contamination (1 ≤ OPI < 2). Furthermore, outfalls B-15, B-16, and B-19 observed as slightly polluted (2 ≤ OPI < 3), outfalls B-1, B-13 to B-14, B-17, B-19, and B-22 to B-24 are found to be moderately polluted (3 ≤ OPI < 4). Outfalls B-2 to B-12 and B-18 are classified as heavily polluted (OPI ≥ 4) (Figure 7B). The source of pollution for this water body includes a variety of pollutants (including household product residues and significant amounts of nitrogen and phosphorus) which are supposedly discharged on a regular basis directly into the river. Indiscriminate discharge from all sorts of sources causes the water quality of all of these rivers to be degrading. Assessment of organic pollution index (OPI) is mainly based on the concentrations of nitrate, nitrite, ammonium, and phosphate. Algae, bacteria, and protozoa require phosphorus for their metabolic development, making it a critical and limiting nutrient in ecosystems (82). Aquatic life is harmed by anthropogenic pollution that contains nitrites, phosphates, and ammonium containing product consumables.

### Characterization Based on Ecological Risk Indices (E_RI_)

Hakanson developed a system for analyzing ecological risks related to toxic response indicators and pollution measurements (39). The Ecological risk indices for the outfall discharge from other peripheral rivers were also evaluated with the information gathered from different studies (83) and presented in Table 4. There is a downward trend in the ecological risk index (E_RI_) for heavy metals assessed along with the Dhaleshwari river's locations, such as D-1 > D-5 > D-2 > D-4 > D-3. The calculated E_RI_ values ranged from 86.09 to 272.6 in the Dhaleshwari River. D-3, which represents the Dhalla (fish market) district, demonstrated the lowest value, while D-1, which represented the central Savar tannery area district, demonstrated the highest value. Due to the tannery's operations in leather and dying industries, there is a substantial danger of ecological destruction. According to Table 4, Outfalls D-1 (Savar tannery), D-5 (Nama Bazar) showed Very high risk (200 ≤ E_RI_ < 300), and Outfalls D-3 (Dhalla, fish market), D-5 (AKS dying) showed Moderate risk (100 ≤ E_RI_ < 150) all of which are indicating a disastrous degree of ecological risk (Table 4). Among the other rivers, Buriganga also observed very high risk (200 ≤ E_RI_ < 300). The ecological risk index was very low (E_RI_ < 100), indicating low risk in the Shitalakshya River, Turag River, and Tongi Canal.

**Table 4:** Ecological risk characterization of the peripheral rivers in Dhaka city.

**Sampling outfall**	** $E_{r}^{i}$ **			***E*_*RI*_**	**Risk grade**
	**Cd**	**Pb**	**Zn**		
D-1	252	19.5	1.098	272.6	Very high risk
D-2	150	3.15	0.32	153.47	Considerable risk
D-3	78	7.65	0.442	86.09	Moderate risk
D-4	126	2.45	0.466	128.92	Moderate risk
D-5	228	9.85	0.858	238.71	Very high risk
Buriganga River	204	17.1	0.63	221.73	Very high risk
Shitalakshya River	15	5.55	0.624	21.174	Low risk
Turag River	48	6.7	0.93	55.63	Low risk
Tongi Canal	21.9	0.12	0.528	22.55	Low risk

At each outfall, there may have been more significant concentrations of Cd, which might explain the higher E_RI_ measurements. The anthropogenic (human-induced) sources of Cd in the environment include phosphate fertilizers, non-ferrous metal mining or refining, and waste disposal (10). A large area of agricultural land surrounds Dhaka, and local people are using most of these lands to cultivate crops. Toxicity levels of Cd have been found in crops and aquatic organisms. Untreated tannery waste, uncontrolled urbanization, raw effluent from various dying businesses, and leather waste along the chosen stretches are specific probable explanations for the ecological disaster.

### Waste Loading Characteristics of Outfalls

**Figure 8:**
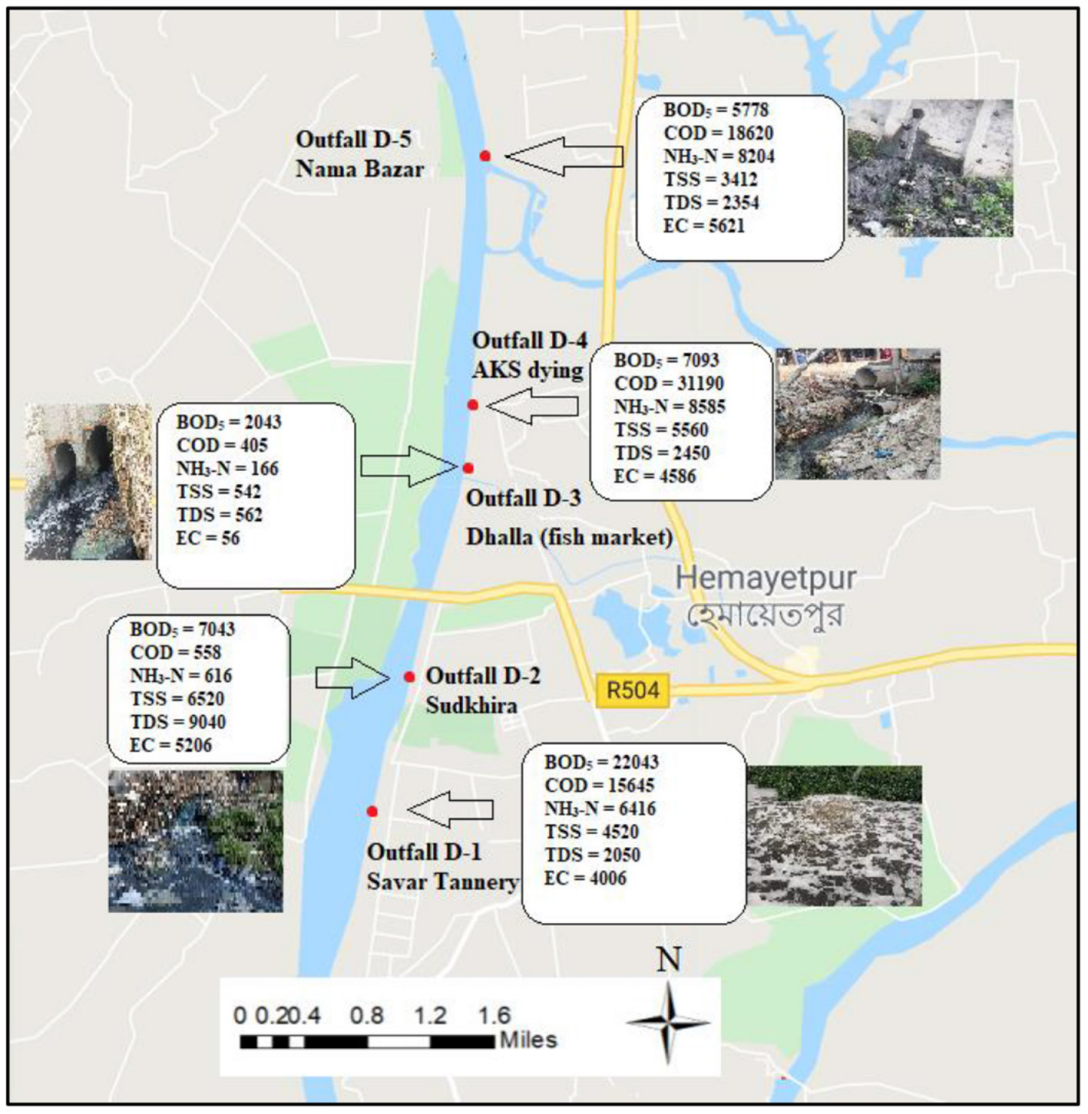
Waste load contribution in Dhaleshwari River.

The present study illustrates the waste loading estimations for the five rivers along with the outfalls locations in the waste loading map from Figure 8 through **Figure 11**. Figure 8 shows waste load contributions in the Dhaleshwari River. The pollution loadings were estimated based on the population density and areas of each drainage catchment from which discharges into the rivers occurred. Drainage network, population figures and unit loading figures were obtained from the Dhaka Water Supply and Sewerage Authority (DWASA) and Browder (84). Outfall D-1 contributes the most toward pollution in the present study from the Savar Tannery area with a BOD_5_ loading rate of 22,043 kg/day, COD loading rate of 15,645 kg/day, NH_3_-N loading rate of 6,416 kg/day, and TDS loading rate of 2,050 kg/day in Dhaleshwari River. Moreover, outfall D-3 could be characterized as the lowest polluted outfall with a BOD_5_ loading rate of 2,043 kg/day, COD loading rate of 405 kg/day, NH_3_-N loading rate of 166 kg/day, and TDS loading rate of 562 kg/day in Dhaleshwari River. Additionally, outfall D-4 also contribute to heavily polluted industrial discharge with a BOD_5_ loading rate of 7,093 kg/day, COD loading rate of 31,190 kg/day, NH_3_-N loading rate of 8,585 kg/day, and TDS loading rate of 2,450 kg/day in Dhaleshwari river.

**Figure 9:**
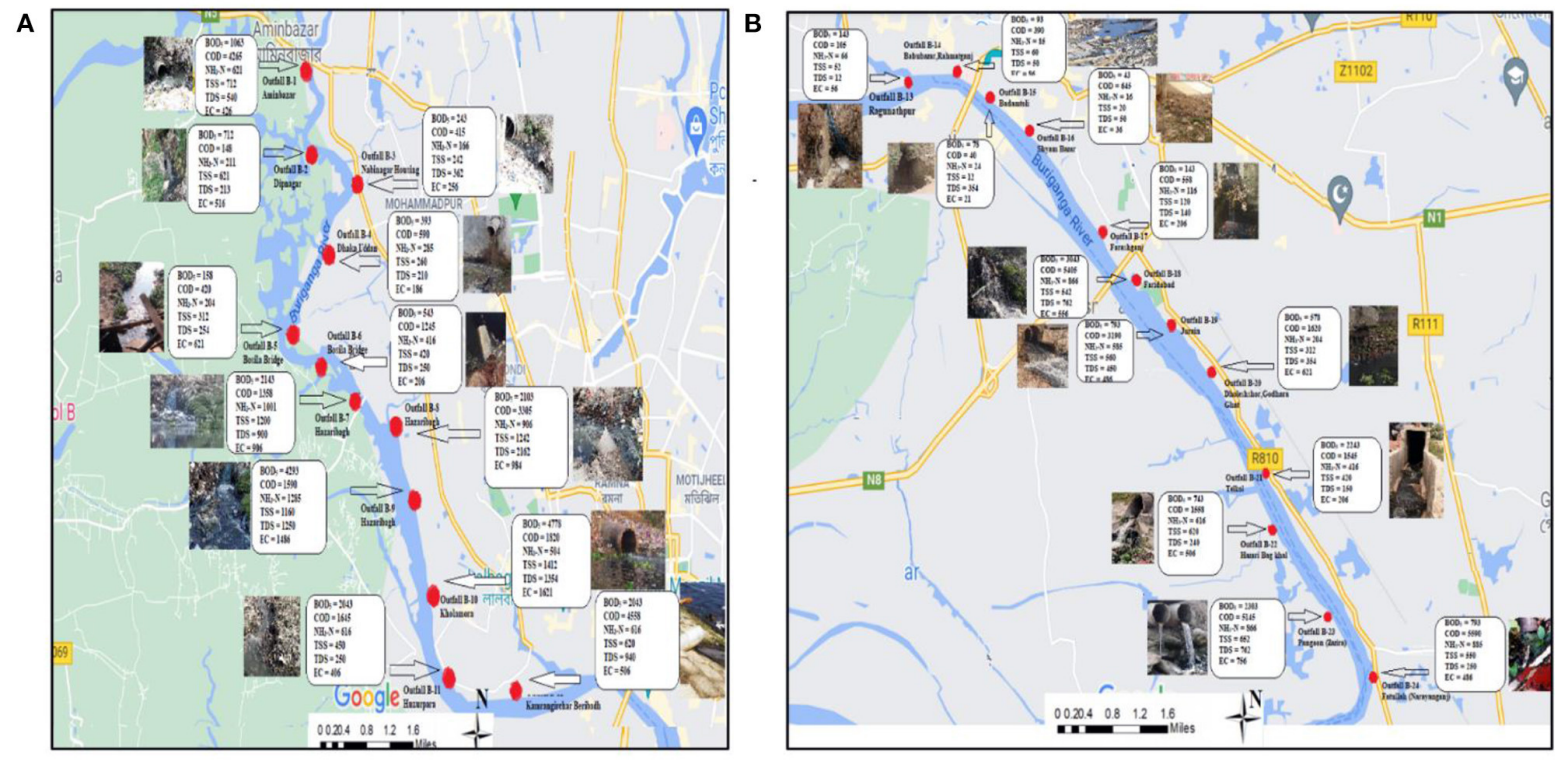
**(A,B)** Waste load contribution in Buriganga River.

Figures 9A,B represent the waste loading map for selected water quality parameters of the Buriganga River from 24 km of selected stretches, along with the outfall locations. Figure 9A depicts the waste loading from outfalls B-1 to B-12, which covered the areas of Aminbazar to Kamrangirchar Beribadh, whereas Figure 9B illustrate the waste loading from outfalls B-13 to B-24 which covered the areas of Ragunathpur to Fatullah (Narayanganj) along the Buriganga river. Outfall B-7 (Hazaribagh) with a BOD_5_ loading rate of 5,143 kg/day, COD loading rate of 1,358 kg/day, NH_3_-N loading rate of 1,001 kg/day, and TDS loading rate of 900 kg/day, contributed the highest loading among all the selected outfalls for Buriganga. Additionally, outfall B-10 (Kholamora) with BOD_5_ loading rate 4,778 kg/day, COD loading rate of 3,305 kg/day, NH_3_-N loading rate of 906 kg/day, and TDS loading rate of 2,162 kg/day, contributed the second-highest loading among all the selected outfalls for Buriganga. Apart from BOD_5_, COD, and TDS are also observed at significant levels between these two outfalls. Outfall B-10 discharged notable amounts of COD (1,820 kg/day), TSS (1,412 kg/day), and ammoniacal nitrogen (504 kg/day) during the study. Outfall B-8 (Hazaribagh) with a TDS loading rate of 2,162 kg/day contributed the highest load for TDS loading among all outfalls along Buriganga. In addition, among the industrial pollution sources, outfall B-24 at Fatullah, Narayanganj released the highest levels of NH_3_-N (885 kg/day) and COD (5,590 kg/day) during the study as shown in Figure 9B along Buriganga. Similar to outfall B-4 (Dhaka Uddan), B-8 (Hazaribagh), B-9 (Hazaribagh) and B-12 (Kamrangirchar Beribadh) contributed significant amounts of NH_3_-N, COD, BOD_5_, and TDS in the Buriganga River. The chemical waste and dye injected from the local textile industries are likely responsible for high concentrations of COD and TSS. Overall, the waste loading data suggested that outfalls B-7 (Hazaribagh) to B-12 (Kamrangirchar Beribadh) constitute the significant pollution route along the Buriganga River.

Apart from that, Alam et al. (43) conducted an outfall study and reported only one outfall from the Hazaribagh area in Buriganga River, contributing as high as 12,245 kg/day of loading rate of BOD_5_ from this individual outfall. From the present study, outfalls B-7, B-8, B-9, and B-10 fall in the same area known as the Hazaribagh area. In comparison, the current study identified four outfalls in total in the same area (B-7 through B-10) and measured BOD_5_ loading rates of 5,143, 4,103, 4,293, and 4,778 kg/day for the outfalls B-7, B-8, B-9, and B-10 (respectively). Therefore, these outfalls should be considered contributing significantly in combination, especially when wastewater sources are discharged into the water next to the river bank directly from the industries. Although tanneries have shifted from Hazaribagh to Savar, there is a significant contribution from the existing and newly emerged outfalls if we consider the whole waste load in combination in the Hazaribagh area.

**Figure 10:**
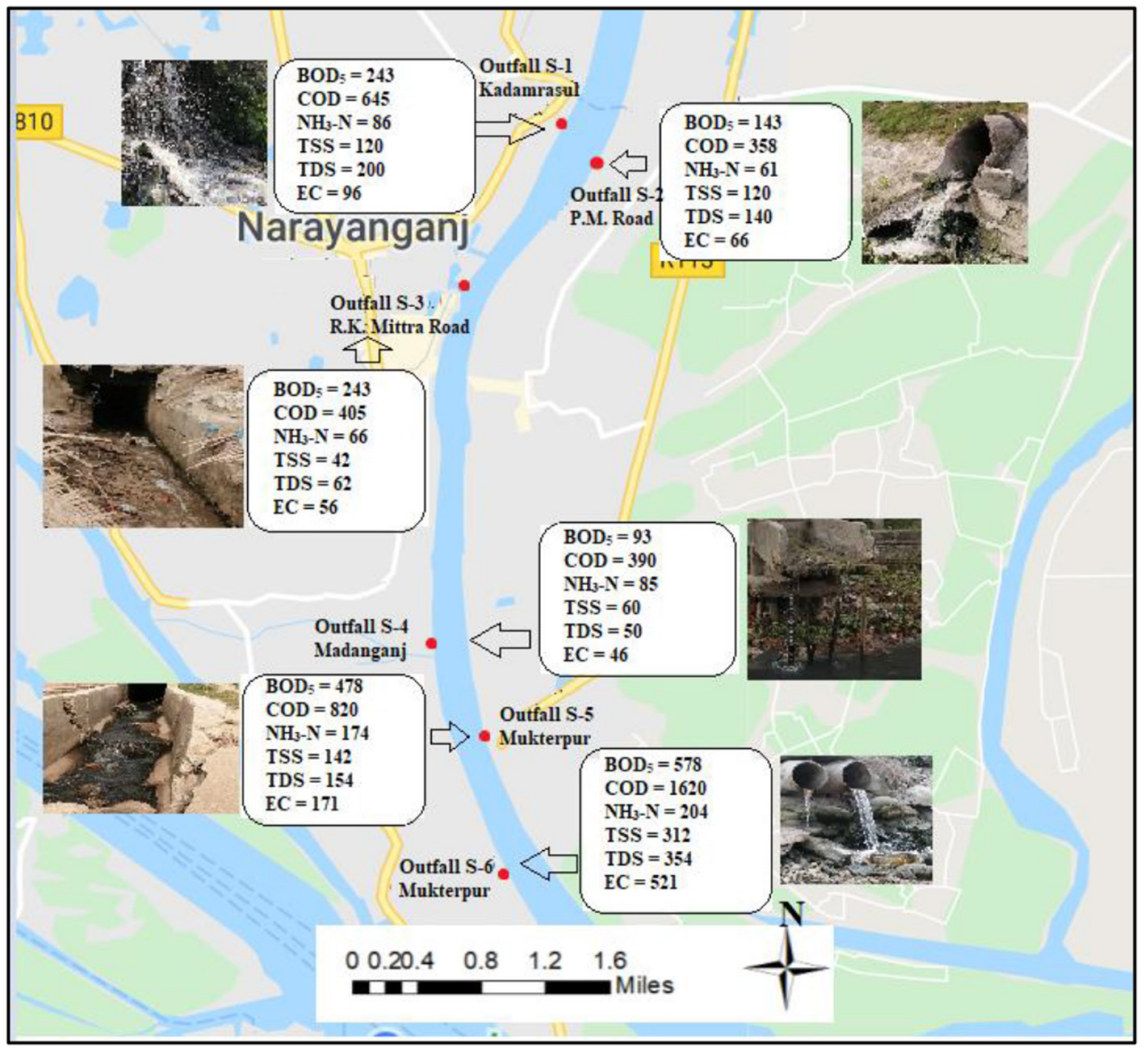
Waste load contribution in Shitalakshya River.

Figure 10 depicts waste load contributions in the Shitalakshya River. Outfall S-6, which is located at Mukterpur, observed the highest load contributed among all outfalls with a BOD_5_ loading rate of 578 kg/day, COD loading rate of 1,620 kg/day, NH_3_-N loading rate of 204 kg/day. In addition, Outfall S-5 which is also located at Mukterpur, observed the second highest load contributed among all outfalls with a BOD_5_ loading rate of 478 kg/day, COD loading rate of 820 kg/day, NH_3_-N loading rate of 174 kg/day. On the other hand, Outfall S-2, which is also located near P.M. Road, observed the lowest load contributed among all outfalls with a BOD_5_ loading rate of 143 kg/day, COD loading rate of 358 kg/day, NH_3_-N loading rate of 61 kg/day.

**Figure 11:**
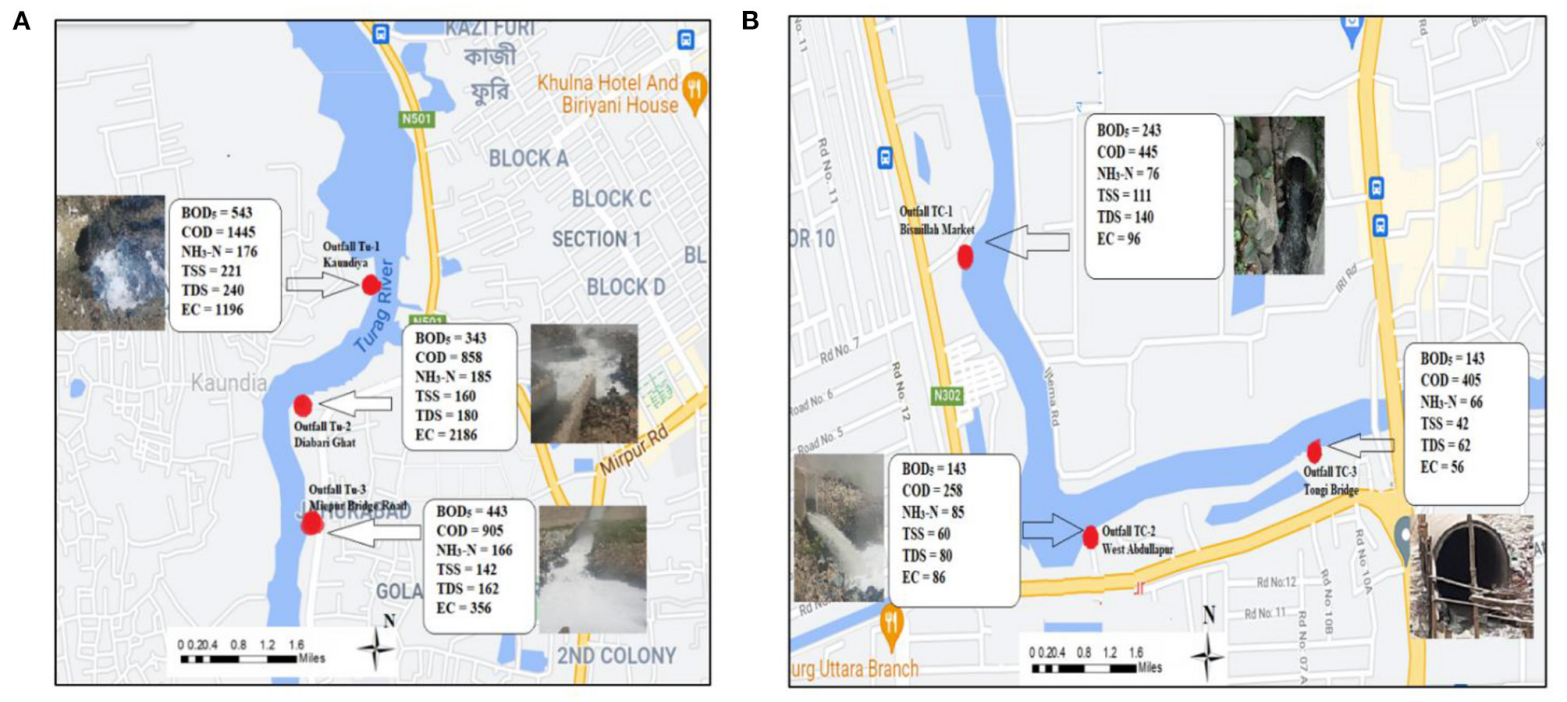
**(A,B)** Waste load contribution in Turag River and Tongi Canal.

Waste loading maps of Turag River and Tongi Canal are illustrated in Figures 11A,B respectively, along with the outfall locations. High-intensity discharge was observed from outfall Tu-3 in Turag River with a BOD_5_ loading rate of 843 kg/day and COD loading rate of 1,505 kg/day. Moreover, outfall Tu-2 also a contributed considerable amount of waste with a BOD_5_ loading rate of 343 kg/day, COD loading rate of 858 kg/day and, TSS loading rate of 160 kg/day. In addition, in Tongi Canal, all the outfalls discharge an adequate amount of waste load from outfall TC-1 and TC-3. Considering above discussed facts, it is to be noted that there are many non-point (diffuse) sources entering the Buriganga-Dhaleswari- Shitalakshya-Tongi Canal-Turag River system, originating either from industries or from domestic wastes. Furthermore, these are causing the accumulation of contaminants into the aquatic ecosystem, which may create severe exposure and relatively high trophic level concentrations.

## Conclusion

Rivers in Dhaka city, the capital of Bangladesh, undergo severe pollution threats due to ever-growing industrial establishments on the banks and indiscriminate discharge from industrial and municipal outfalls. This study performed and demonstrated comprehensive identification of outfalls along the outlying rivers of Dhaka city to highlight the pollution density along the rivers. The study also dealt with assessments of subsequent pollution status and aquatic ecological threats due to organic and inorganic water contaminants and heavy metals such as cadmium, lead and zinc that are discharged from the outfalls along the selected stretches of the peripheral rivers around the Dhaka watershed. Significant contamination with respect to dissolved solids and organic content was evident at each of the peripheral rivers. The concentration levels of the toxic metals in outfalls of the Savar tannery, Dhalla fish market AKS dying and Nama Bazar (D-5) areas of Dhaleshwari River, Turag River, and Buriganga river, in general, seemed to be of significant and of grave concern warranting regular and detailed investigation and monitoring. The Characterization based on Comprehensive and Organic Pollution Indices for each sampling outfalls of all the rivers in Dhaka Watershed confirmed severely polluted and heavily contaminated water. Ecological risk indices indicated comparatively Lower risk at Shitalakhshya and Tongi canal, Considerable risk at Turag, Very high risk at Dhaleshwari and Disastrous level of risk at Buriganga river. Furthermore, the waste loading estimation indicated that the outfalls located along the selected stretches of Dhaleshwari River, Amin Bazer, Hazaribagh and Faridabad area from Burganga River and Mukterpur area from Shitalakshya River contributed as the primary pollution sources. Substantial industrial waste was also released downstream near Tongi Canal and Turag River.

Surface discharge quality from outfalls, toxicity-based risk characterization and the observations for wastewater discharge revealed that nearby sources significantly impact the characteristics of surface water quality in Dhaka Watershed. If appropriate measures are not adopted soon enough, this will impact the river's ecological health with consequences toward public health. The ultimate solution to prevent the current pollution level along the river involves adequate coverage of sewer network, wastewater treatment and management. In the context of ever-increasing industrial expansion and urbanizations in Dhaka City, current research lays down the foundations for the regular monitoring of the river systems and effluents that it assimilates from the outfalls. Regular assessments of waste disposal amounts and pollutant loading contributions are required periodically in order to formulate strategies to mitigate the water pollution in Dhaka Watershed.

## Data Availability Statement

The data that support the findings of this study are available from [Department of Civil Engineering, University of Asia Pacific], but restrictions apply to the availability of these data, which were used under license for the current study, and so are not publicly available. However, data are available from the authors upon reasonable request and with permission of [Department of Civil Engineering, University of Asia Pacific].

## Author Contributions

MI and NM: conceptualization, investigation, and writing—review and editing. MI: data collection, analysis, and writing—original draft preparation. NM: supervision. All authors have read and agreed to the published version of the manuscript.

## Funding

This work was supported by the undergraduate thesis fund of the department of Civil Engineering, University of Asia Pacific, Dhaka, Bangladesh.

## Conflict of Interest

The authors declare that the research was conducted in the absence of any commercial or financial relationships that could be construed as a potential conflict of interest.

## Publisher's Note

All claims expressed in this article are solely those of the authors and do not necessarily represent those of their affiliated organizations, or those of the publisher, the editors and the reviewers. Any product that may be evaluated in this article, or claim that may be made by its manufacturer, is not guaranteed or endorsed by the publisher.
