# Supplementary material for: Contaminant Discharge From Outfalls and Subsequent Aquatic Ecological Risks in the River Systems in Dhaka City: Extent of Waste Load Contribution in Pollution
Source: Front Public Health. 2022 May 26;10:880399. doi: 10.3389/fpubh.2022.880399 (PMC9177986; doi:10.3389/fpubh.2022.880399)
Supplement: Supplementary file 1 [file Data_Sheet_1.docx]

Supplementary Material

Title: Contaminant Discharge from Outfalls and Subsequent Aquatic Ecological Risks in The River Systems in a Capital: Extent of Waste Load Contribution in Pollution

# Supplementary Tables

## Identification of sampling outfalls and name of area

| **Name of River** | **Sampling Outfalls** | **Type of Discharge** | **Name of Area** |
| --- | --- | --- | --- |
| Dhaleshwari River | D-1 | Industrial | Savar Tannery |
|  | D-2 | Municipal | Sudkhira |
|  | D-3 | Industrial | Dhalla (fish market) |
|  | D-4 | Industrial | AKS dying |
|  | D-5 | Industrial | Nama Bazar |
| Buriganga River | B-1 | Industrial | Aminbazar |
|  | B-2 | Industrial | Dipnagar |
|  | B-3 | Industrial | Nabinagar Housing |
|  | B-4 | Mixed | Dhaka Uddan |
|  | B-5 | Industrial | Bosila Bridge |
|  | B-6 | Municipal | Bosila Bridge |
|  | B-7 | Industrial | Hazaribagh |
|  | B-8 | Combined Industrial | Hazaribagh |
|  | B-9 | Combined Industrial | Hazaribagh |
|  | B-10 | Combined Industrial | Kholamora |
|  | B-11 | Combined Industrial | Huzurpara |
|  | B-12 | Industrial | Kamrangirchar Beribadh |
|  | B-13 | Municipal | Ragunathpur |
|  | B-14 | Municipal | Babubazar,Rahmatganj |
|  | B-15 | Municipal | Badamtoli |
|  | B-16 | Municipal | Shyam Bazar |
|  | B-17 | Municipal | Farashganj |
|  | B-18 | Industrial | Faridabad |
|  | B-19 | Municipal | Jurain |
|  | B-20 | Industrial | Dholeshshor,Godhara Ghat |
|  | B-21 | Municipal | Telkol |
|  | B-22 | Industrial | Hazari Bag khal |
|  | B-23 | Industrial | Pangaon (Zazira) |
|  | B-24 | Industrial | Fatullah (Narayanganj) |
| Shitalakshya River | S-1 | Industrial | Kadamrasul |
|  | S-2 | Municipal | P.M. Road |
|  | S-3 | Industrial | R.K. Mittra Road |
|  | S-4 | Industrial | Madanganj |
|  | S-5 | Municipal | Mukterpur |
|  | S-6 | Industrial | Mukterpur |
| Turag River | Tu-1 | Industrial | Kaundiya |
|  | Tu-2 | Industrial | Diabari Ghat |
|  | Tu-3 | Industrial | Miepur Bridge Road |
| Tongi Canal | TC-1 | Industrial | Bismillah Market |
|  | TC-2 | Municipal | West Abdullapur |
|  | TC-3 | Industrial | Tongi Bridge |

## Classifications of CPI categories (Mishra et al., 2016)

| CPI | Classification |
| --- | --- |
| 0 < CPI < 0.2 | Clean |
| 0.2 < CPI < 0.4 | Sub clean |
| 0.4 < CPI < 1 | Slightly polluted |
| 1 < CPI < 2 | Medium polluted |
| CPI ≥ 2 | Severely polluted |

## Classifications of OPI categories (Al-Aboodi et al., 2018)

| **OPI** | **Classification** |
| --- | --- |
| 0 < OPI | Excellent |
| 0 ≤ OPI < 1 | Good |
| 1 ≤ OPI < 2 | Water is beginning to be contaminated |
| 2 ≤ OPI < 3 | Lightly polluted |
| 3 ≤ OPI < 4 | Moderately polluted |
| OPI ≥ 4 | Heavily polluted |

## Classification of Ecological risk index, and E_RI_ of heavy metal pollution (Biswas et al., 2015).

| **E^i^_r_** | **E_RI_** | **Classification** |
| --- | --- | --- |
| E^i^_r_ < 30 | E_RI_ < 100 | Low risk |
| 30 ≤ E^i^_r_ < 50, | 100 ≤ E_RI_ < 150 | Moderate risk |
| 50 ≤ E^i^_r_ < 100 | 150 ≤ E_RI_ < 200 | Considerable risk |
| 100 ≤ E^i^_r_ < 150 | 200 ≤ E_RI_ < 300 | Very high risk |
| E^i^_r_ ≥ 150 | E_RI_ ≥ 300 | Disastrous risk |

## Results of Water Quality Parameters of the Peripheral Rivers of Dhaka City:

| **Parameter** | **Unit** | **Standard^*^** | **Average ± Standard Error of Mean** | | | | |
| --- | --- | --- | --- | --- | --- | --- | --- |
|  |  |  | **Dhaleshwari River (n=5)** | **Turag River (n=3)** | **Tongi Canal (n=3)** | **Buriganga River (n=24)** | **Shitalakshya River (n=6)** |
| Nitrate | mg/L | 1 | 2.14 ± 1.45 | 0.36 ± 0.1 | 0.31 ± 0.03 | 0.48 ± 0.19 | 3.05 ± 1.1 |
| Nitrite | mg/L | 10 | 19.33 ± 12.8 | 0.2 ± 0.002 | 0.4 ± 0.005 | 0.4 ± 0.2 | 0.2 ± 0.01 |
| Phosphate | mg/L | 6 | 55.05 ± 5.13 | 18.06 ± 3.01 | 15.7 ± 1.2 | 3.18 ± 0.8 | 40.5 ± 12.3 |

*Standards for waste from industrial units or projects waste: The environment conservation rules, Bangladesh, 1997 (ECR’97), n = Number of outfalls

# Supplementary Figures

## Selected stretches of Buriganga River with identified and selected outfalls along the river.


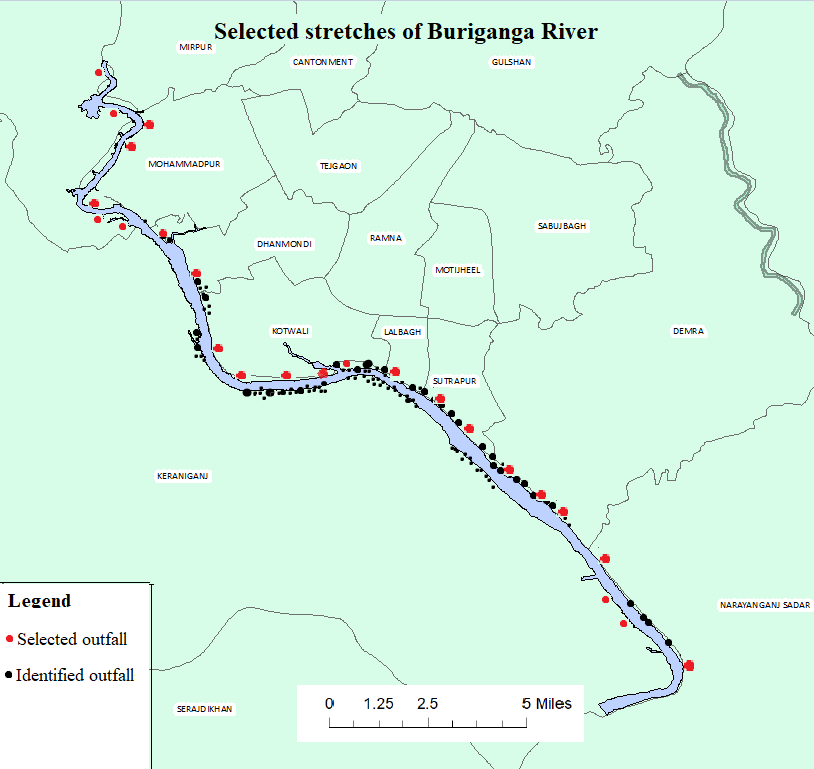


## Selected stretches of Dhaleshwari River with identified and selected outfalls along the river.


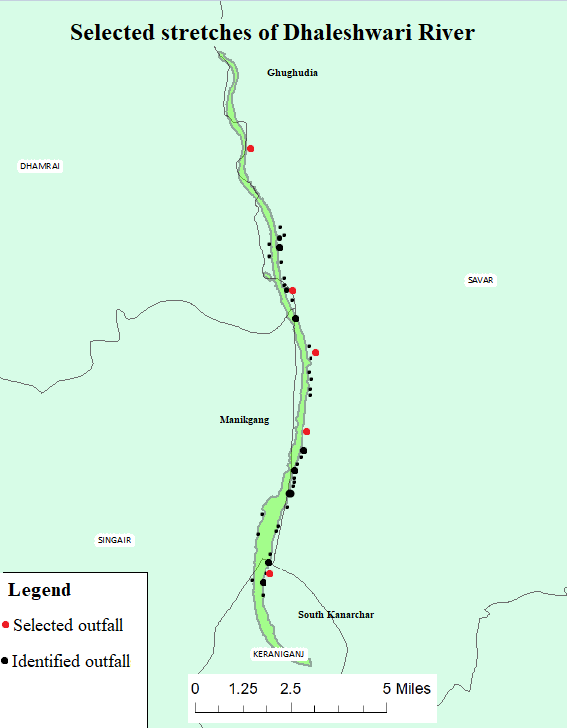


## Selected stretches of Shitalakshya River with identified and selected outfalls along the river.


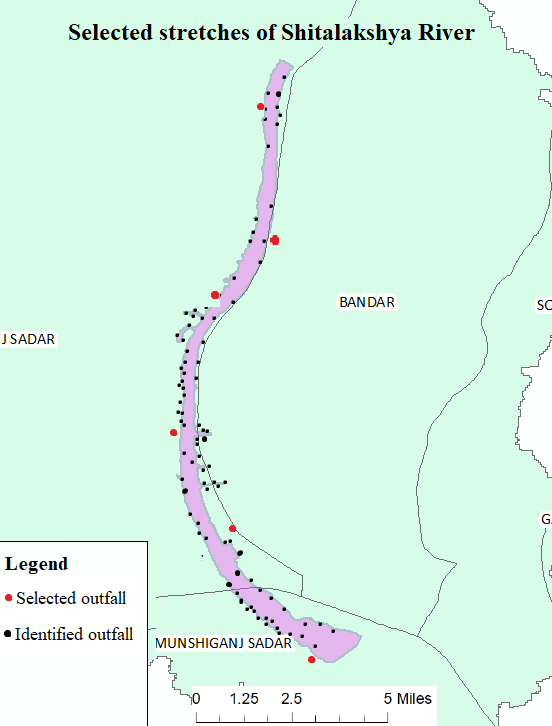


## Selected stretches of Tongi Canal with identified and selected outfalls along the river.


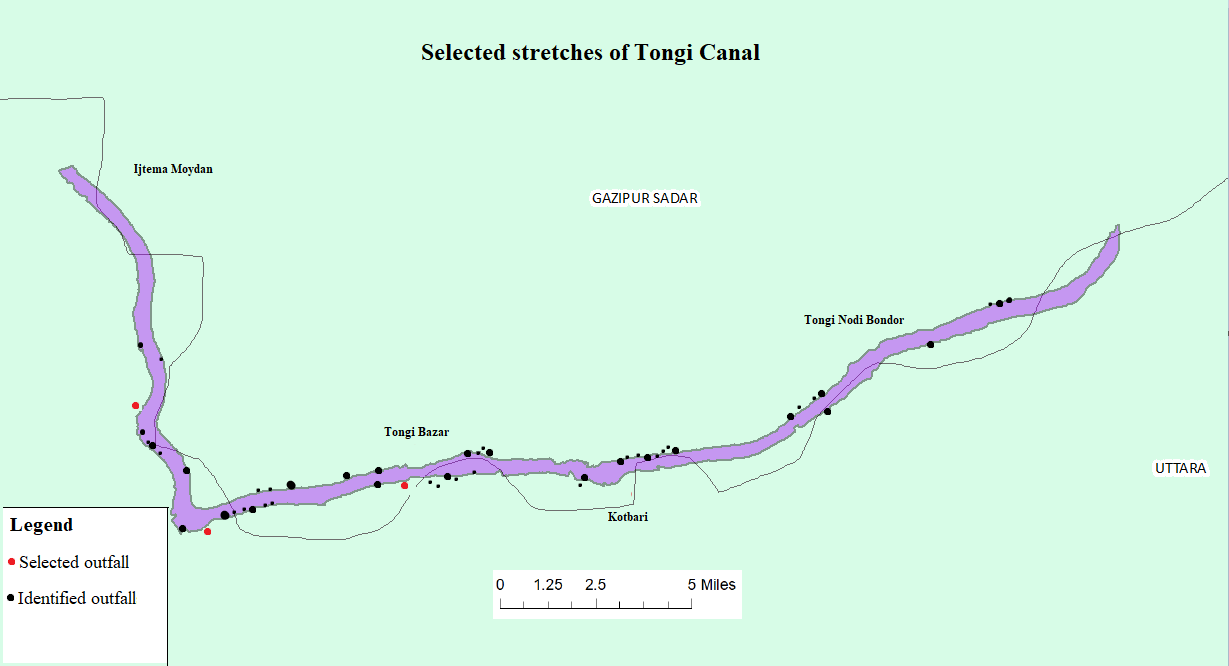


## Selected stretches of Turag River with identified and selected outfalls along the river.


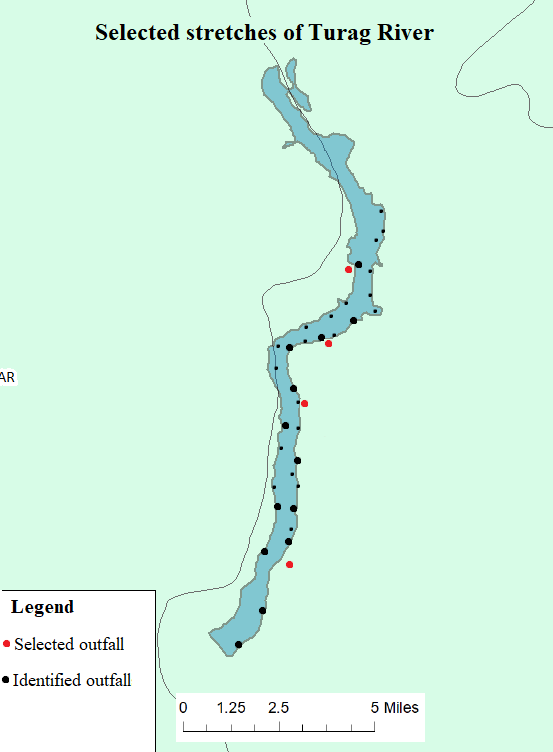


**2.6 Identified outfalls in Dhaka Watershed**

**
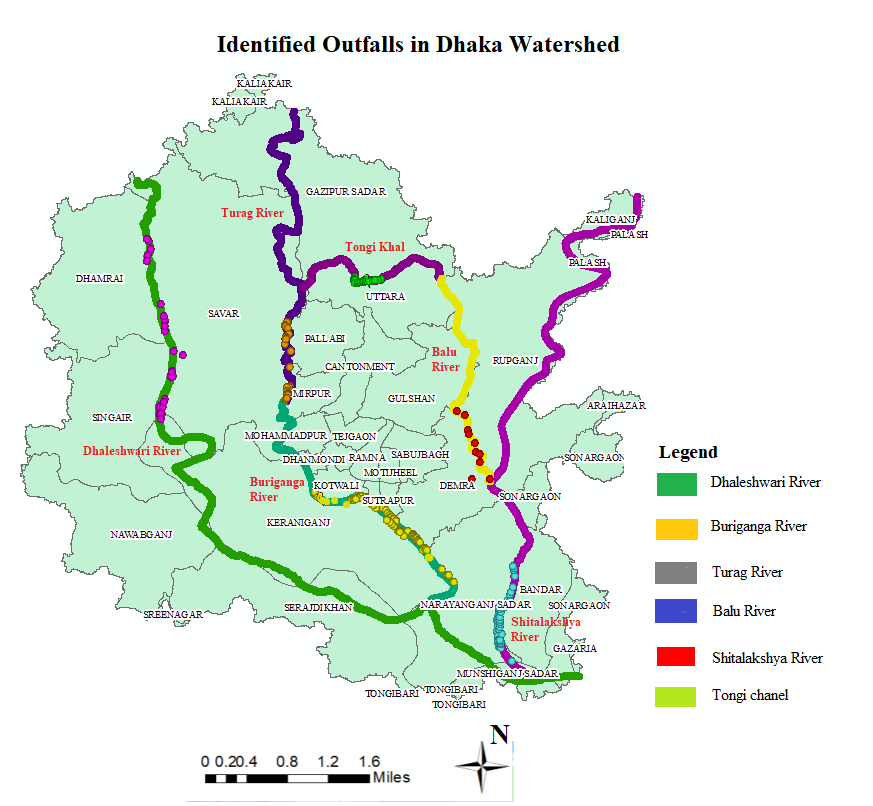
**
